# Supplementary material for: Elder abuse as a risk factor for psychological distress among older adults in India: a cross-sectional study
Source: BMJ Open. 2017 Oct 22;7(10):e017152. doi: 10.1136/bmjopen-2017-017152 (PMC5665217; doi:10.1136/bmjopen-2017-017152)
Supplement: Author's manuscript [file bmjopen-2017-017152.draft_revisions.pdf]

BMJ Open is committed to open peer review. As part of this commitment we make the peer review history of every article we publish publicly available.

When an article is published we post the peer reviewers' comments and the authors' responses online. We also post the versions of the paper that were used during peer review. These are the versions that the peer review comments apply to.

The versions of the paper that follow are the versions that were submitted during the peer review process. They are not the versions of record or the final published versions. They should not be cited or distributed as the published version of this manuscript.

BMJ Open is an open access journal and the full, final, typeset and author-corrected version of record of the manuscript is available on our site with no access controls, subscription charges or pay-per-view fees (http://bmjopen.bmj.com).

If you have any questions on BMJ Open's open peer review process please email editorial.bmjopen@bmj.com

BMJ Open

Elder abuse as a risk factor for psychological distress among older adults in India: a cross-sectional study

Journal:	*BMJ Open*
Manuscript ID	bmjopen-2017-017152
Article Type:	Research
Date Submitted by the Author:	05-Apr-2017
Complete List of Authors:	Evandrou , Maria ; University of Southampton, Centre for Research on Ageing, ESRC Centre for Population Change<br>Falkingham, J; University of Southampton<br>Qin, Min; Centre for Research on Ageing, ESRC Centre for Population Change<br>Vlachantoni, Athina; University of Southampton
**Primary Subject Heading**:	Public health
Secondary Subject Heading:	Mental health
Keywords:	Elder abuse, Risk factors, MENTAL HEALTH

SCHOLARONE™ Manuscripts

For peer review only

Elder abuse as a risk factor for psychological distress among older adults in India: a cross-sectional study

Maria Evandrou[1], Jane Falkingham[1], Min Qin[1] and Athina Vlachantoni[1]

[1]Centre for Research on Ageing and ESRC Centre for Population Change, University of Southampton, Southampton, SO17 1BJ, UK

Correspondence to

Dr. Min Qin

Centre for Research on Ageing and ESRC Centre for Population Change

University of Southampton

Southampton, SO17 1BJ

UK

Tel +44 (0)23 80 594290

Fax +44 (0)23 80 594290

Email: min.qin@soton.ac.uk

Word count 4541

For peer review only

For peer review only - http://bmjopen.bmj.com/site/about/guidelines.xhtml

Abstract

Objectives This study examines the association between elder abuse and psychological distress among older adults in India, and explores whether this association varies by the level of psychosocial and material resources.

Methods Bi-variate and multivariate analyses were conducted using data on a representative sample of 9,692 adults aged 60 and above in seven Indian states from the UNFPA project 'Building Knowledge Base on Ageing in India (BKPAI)'. Any reported elder abuse (physical and/or emotional) from family members one month before the survey was examined.

Results The results show that the experience of abuse is negatively associated with the mental health of older adults, and this relationship persists even after controlling for demographic and socio-economic factors. The findings also suggest that household wealth has an inverse relationship with mental health, with the association between experiencing elder abuse and reporting poor mental health being strongest amongst older people in wealthy households.

Conclusions Elder abuse in India is currently a neglected phenomenon, and greater recognition of the link between abuse and mental health is critical in order to improve the well-being of vulnerable older adults, some of whom may be 'hidden' within well-off households.

Key Words: Elder abuse—Risk factors—Mental health

For peer review only

For peer review only - http://bmjopen.bmj.com/site/about/guidelines.xhtml

Strengths and limitations of this study

- The findings of the study are from a large representative sample of 9692 older adults from selected Indian states.
- Abuse data in this study were self-reported and there was no validation by agencies charged with investigating elder abuse.
- It is possible that older people already had stress before the experience of abuse, and at the time of the interview were more likely than others to recall past experiences such as abuse.

For peer review only

For peer review only - http://bmjopen.bmj.com/site/about/guidelines.xhtml

Introduction

India's population is ageing. With improvements in mortality as a result of rising living standards, improved sanitation, public health and medical advances, more people are living longer and surviving into old age. Such trends, combined with recent falls in fertility, mean that the number of older people is both increasing in absolute terms and also as a share of the population. In 1980, individuals aged 60 and over accounted for just 5.9 percent of the Indian population; by 2015 this had risen to 8.9 percent, comprising 116.5 million people, and by 2050 older people are projected to constitute nearly one fifth (19.4%) of the total population, with 330 million Indians in their sixties or older. [1] The changes in the age structure of India's population are being accompanied by other social and economic transformations including rapid urbanization and industrialization. Increasing women's participation in paid employment, greater internal and international mobility amongst the younger generation and the growth of individualism are all impacting upon the traditional Indian family system; a system which has emphasized the obligation of sons and their wives to respect, obey, and provide care for their aged parents. [2]

Traditionally, elders have been respected in Indian society, and families are the principal financial, emotional and physical caregivers for older relatives. [3] Although this tradition remains today, [4] qualitative studies have demonstrated that both respect for older people and the caring traditions of the extended family are on the wane in the larger context of societal and cultural changes. Older people are more likely to be exposed to abuse, isolation and abandonment. [5-6] Elder abuse is internationally defined as a "single, or repeated act, or lack of appropriate action, occurring within any relationship where there is an expectation of trust

For peer review only

which causes harm or distress to an older person". [7] Elder abuse can manifest as physical, emotional, sexual, and financial abuse, and/or as intentional or unintentional neglect. Qualitative research has found that selected later life mental disorders may be attributed to abuse, neglect, or a lack of love from children. [5] However, psychological distress in later life remains an under-researched area in India, particularly in terms of the psychological consequences associated with elder abuse and neglect. [8-9] This study aims to contribute to the literature by investigating the association between elder abuse and psychological distress among older adults in India and examining whether this association varies by the level of psychosocial and material resources at older adults' disposal.

The relationship between elder abuse, resources and psychological distress

Psychological distress comprises of a variety of symptoms such as depression, anxiety, stress and insomnia. The level of such distress experienced by an individual at any point in time is determined by various biological and psychosocial factors. [10-11] Elder abuse is a stressful experience which has been found to have harmful effects on mental health, [12] with depression, anxiety and post-traumatic disorder being reported as the most prevalent psychological consequences. [13-14]

The general stress theory postulates that the effect of stressors (i.e. stressful events) on psychological health operates in three phases: alarm, resistance, and exhaustion, and is a process that involves changes in individuals' immune system, endocrine system and cardiovascular reactivity. [15] When problems accumulate, persist and strain individuals, then

For peer review only - http://bmjopen.bmj.com/site/about/guidelines.xhtml

adaptation resources are depleted and a stimulated parasympathetic system may lead to worrying, anxiety, depression, anger and/or other physical illness. Studies have found that older adults who are mistreated have higher levels of psychological distress than those who have no such experience. [16-17] The frequency or type of elder abuse also has an impact on mental health. Fisher and Regan (2006) [18] found that repeated abuse or multiple types of elder abuse (e.g. emotional) were risk factors for depression or anxiety among older women. Based on previous empirical findings, this paper hypothesizes (H1) *that older adults who report experiencing elder abuse will have higher odds of psychological distress than those who do not report such experiences*.

However, many individuals who experience stressful events do not go on to develop a psychological illness. The stress-buffering model suggests that psychosocial resources moderate the deleterious effects of high levels of stress. The statistical interaction between stress and resources can be used to test these moderating effects. [19] Resources may intervene between the experience of stress and the onset of mental illness by providing a solution to the problem or reducing the perceived importance of the problem, which in turn helps to decrease or eliminate the stress reaction. Psychosocial resources that can buffer the negative impact of life events on psychological well-being include subjective resources such as high self-esteem, mastery, social support and social participation, and objective resources such as socioeconomic status, including income and household wealth.[20-22] For instance, a beneficial effect of social support on one's mental health could occur thanks to large social networks providing individuals with regular positive experiences and stable, socially-rewarding roles in the community. This kind of support could provide a positive effect, a sense of predictability

For peer review only - http://bmjopen.bmj.com/site/about/guidelines.xhtml

and stability in one's life situation, and recognition of self-worth, all of which are related to overall well-being. At the same time, material resources such as income and household wealth can offer protection against negative experiences associated with economic problems. Indeed, previous empirical studies have found that in the presence of stress from elder abuse, supportive relationships may buffer the effect of stress. [23,17] As such, this paper hypothesizes that (H2) *the negative association of elder abuse with psychological distress will be stronger for those with fewer psychosocial and material resources than for those with more psychosocial and material resources*.

Data and methods

This study analyses data collected as part of the UNFPA 'Building Knowledge Base on Ageing in India (BKPAI)' project. The BKPAI Survey was conducted in 2011 in seven major demographically advanced states of India - Himachal Pradesh, Punjab, West Bengal, Odisha, Maharashtra, Kerala and Tamil Nadu. A representative sample was obtained using a random sampling method covering the Northern, Southern, Western and Eastern regions. The detailed information about the survey sampling is described in a previous report. [24] The primary sampling units were households. All those aged 60 and above in the sampled households were interviewed face-to-face. The completion rate for households was 94.7 percent and 92.9 percent for elderly respondents. Non-response at both the household and individual levels was adjusted through the sampling weights calculation by the research organisation. The BKPAI survey data includes information on older people's mental and physical health, their

For peer review only - http://bmjopen.bmj.com/site/about/guidelines.xhtml

For peer review only

living arrangements, socio-economic circumstances, including employment status and household assets, as well as information on intergenerational exchanges within the family and participation in social activities. The total sample size interviewed is 9,692. Of these, 103 are excluded because of missing values (missingness is not mutually exclusive) on psychological distress (N=36); education (N=53); whether has someone for trust/confidence (N=7); and whether feels able to manage unexpected situations (N=16). The final analytical sample is 9,589 adults aged 60 and above.

Measurements

Psychological distress

The 12-item version of the General Health Questionnaire (GHQ-12) is used as a measure of psychological distress. These questions have been widely used to identify minor psychiatric disorders in the general population. [25] The GHQ-12 has been previously validated in India in clinical surveys conducted in Kannada, [26-27] Hindi [28] and Tamil. [29] Given that the BKPAI study was conducted across multiple states with different languages and focused amongst older adults, it was important for the team to test the reliability of GHQ-12 within the BKPAI. The measure was found to have high internal consistency with an overall Cronbach's alpha of 0.9. Examining each state individually, Cronbach's alpha ranged from a low of 0.7 in West Bengal to a high of 0.94 in Himachal Pradesh, suggesting that the measure may be considered to be valid across all seven states and in all the languages used.

For peer review only - http://bmjopen.bmj.com/site/about/guidelines.xhtml

Using the standard GHQ scoring method, the four category responses for each of the 12 questions are coded (0, 0, 1, 1), with the points summed to produce a total score ranging from 0-12. We used a score of >=4 as the threshold to define psychological distress according to studies validating the GHQ-12 against standardized psychiatric interviews. [30,27]

Elder abuse

In the BKPAI survey, the respondents were asked two sets of questions regarding their experience of abuse since they were 60 years old and in the last month. The first question was *'In the time since you completed 60 years of age have you faced any type of abuse or violence or neglect or disrespect by any person?'* If the respondent answered 'Yes', a follow-up questions asked the type of abuse (Physical abuse, Verbal abuse, Economic abuse, Showing disrespect, Neglect and Other) and where it originated (within family, outside family, both within family & outside family). *A further question asked 'Have you faced any type of physical or emotional abuse or violence in the last month?'* The responses include '*1. No, 2. Physical, 3. Emotional, 4. Both physical and emotional*'. All other types of violence other than physical were merged into emotional violence. If the respondent answered in the affirmative, follow-up questions elicited the source of abuse which could include '*1. Spouse, 2. Son, 3. Daughter, 4. Son-in-law, 5. Daughter-in-law, 6. Domestic helper, 7. Grandchildren, 8. Relatives, 9. Neighbours, 10. Other*'. Careful attention was paid to avoid the appearance of any family members during this particular part of data collection and to guarantee the confidentiality of information. [24]

For peer review only - http://bmjopen.bmj.com/site/about/guidelines.xhtml

For peer review only

A previous study based on this data reported that 11 per cent of respondents have experienced at least one type of abuse *after the age of 60*. Verbal abuse is most frequently claimed, followed by disrespect, economic abuse, neglect and physical abuse, with the most common perpetrator being the respondent's son. [9] In this study we concentrate on older adults who report having experienced physical and/or emotional abuse in *the last month*, distinguishing between those who report abuse by family members and others, to examine the contemporaneous interaction between elder abuse, psychosocial and material resources, and psychological distress. Here, abuse is limited to that reported as emanating from family members as it is this form of abuse that we hypothesise may have increased as a result of recent changes impacting the traditional Indian family system.

Psychosocial and material resources

An individual's psychosocial resources include personal qualities such as optimism, psychological control or mastery, and self-esteem, as well as the availability of social support, all of which can help to manage stressful events and contribute to better health outcomes. [31,20,22] There are a variety of scales measuring social support and personal coping resources. [32-33] The BKPAI was not explicitly designed to measure psychosocial resources. It does, however, contain a number of important indicators of potential support and coping resources, including being married or living together with one's partner, participation in social activities, having someone to trust and confide in, and feeling able to manage unexpected situations.

For peer review only - http://bmjopen.bmj.com/site/about/guidelines.xhtml

For peer review only

Participation in social activities is defined as having participated in any of the five listed activities in the last 12 months: attending a public meeting with discussion of local, community or political affairs; attending any group, club, society, union or organizational meeting; working with other people in your neighbourhood to fix or improve something; attending or participating in any religious programs/services (not including weddings and funerals); going out of the house to visit friends or relatives.

The question on *feeling able to manage unexpected situations* has three response categories: most of the time, sometimes, and hardly ever feeling that one can manage situations even when they do not turn out to be as expected.

Material resources include personal sources of income (no income, one source, multiple sources) and household wealth quintile. Household wealth quintile is computed using principle component analysis (PCA) based on 30 assets and housing characteristics: household electrification; drinking water source; type of toilet facility; type of house; cooking fuel; house ownership; ownership of a bank or post-office account; and ownership of a mattress, pressure cooker, chair, cot/bed, table, electric fan, radio/transistor, black and white television, colour television, sewing machine, mobile telephone, any landline phone, computer, internet facility; refrigerator, watch or clock, bicycle, motorcycle or scooter, animal-drawn cart, car, water pump, thresher and tractor. This measure was found to provide a good socio-economic gradient of health outcomes among older adults in the survey. [24]

Other control variables

For peer review only - http://bmjopen.bmj.com/site/about/guidelines.xhtml

Covariates include the individual's age group, sex, education, caste, working status, living arrangement, self-reported health, chronic disease, health-related limitations to daily activities, disability and geographic factors (rural/ urban residence, and state). An individual's difficulty with Activities of Daily Living (ADL) is computed based on the level of independence reported by the older person in carrying out the activities of feeding, bathing, dressing, toilet, mobility and continence. Each question has three response categories: 'Do not require assistance; Require partial assistance; Require full assistance'. These are scored as 0, 1 or 2 respectively and are then summed across the six questions, resulting in a total score ranging between 0-12. Given the unequal intervals between the score, rather treating it as a continuous variable, we group it into an ordered categorical variable. Older respondents are defined as having 'no need for assistance' if the total score is 0, as having a 'light need for assistance' if the total score is between 1-5, and as having a 'heavy need for assistance' if the total score is ≥ 6.

Disability is computed based on the respondents' level of reported ability to see, hear, walk, chew, speak and remember. Each question has three response categories 'Yes fully, Yes partially, No'. These are scored as 0, 1 or 2 respectively and are then summed across the six questions, resulting in a total score of 0-12. Again, because of the unequal intervals between the score, we group the total score into an ordered categorical variable. Older people are defined as having 'no disability' if the total score is 0, 'light disability' if the total score is between 1-2, 'medium disability' with a score of 3-4, and 'high disability' if the total score is ≥ 5.

For peer review only - http://bmjopen.bmj.com/site/about/guidelines.xhtml

Method

A series of logistic regression models are estimated with the dependent variable being the report of psychological distress (GHQ ≥ 4 contrast to GHQ ≤ 3). The first model estimates the bivariate association between elder abuse and psychological distress. The second model adds the measures of psychosocial and material resources and other control variables to estimate the association of elder abuse and psychosocial and material resources, with psychological distress after controlling for other covariates. The final model includes the interaction terms of elder abuse with psychosocial and material resources variables to the main-effects-only model to examine whether psychosocial and material resources buffer the association between elder abuse and psychological distress.

Results

Descriptive findings

Table 1 presents descriptive statistics for the total analytical sample. The overall prevalence of psychological distress amongst persons aged 60 and over living in the seven Indian States is 40.6 per cent. Around 5 per cent of older adults had experienced some form of physical or emotional abuse or violence in the last month. Among this sub-group, the prevalence of psychological distress is much higher than in the general older population, at 61.6 per cent.

The indicators of psychosocial resources and socio-economic status appear to have an inverse relationship with psychological distress, with those older people living in households in the

For peer review only - http://bmjopen.bmj.com/site/about/guidelines.xhtml

highest wealth quintile having a prevalence rate of 21.4 per cent compared to 64.1 per cent amongst those living in households in the lowest wealth quintile. Similarly, those who participated in social activities in the last month are less likely to experience psychological distress than those who did not (37 v. 55.8 per cent respectively).

Indicators of health status including self-related health, difficulty with ADLs and disability all show a positive association with psychological distress. Older people living with their spouse only experience the lowest prevalence of psychological distress (34.9 per cent), while those living alone show the highest prevalence (50.9 per cent). Levels of psychological distress increase with age and are higher among older women (44.7 per cent) than older men (36.2 per cent). One's place of residence seems to play an important role with elders living in urban areas having a lower level of psychological distress than their rural counterparts (35.1 v. 45.7 per cent). There are also considerable inter-state variations in the prevalence of psychological distress. A relatively low level of psychological distress is found among older adults in Punjab (20.8 per cent) and Himachal Pradesh (23.5 per cent), contrasting with much higher levels in West Bengal (60.5 per cent) and Odisha (55.5 per cent).

For peer review only

Multivariate analysis results

Table 2 shows the odds ratios from the logistic regression models. Model 1 shows the simple bi-variate relationships between elder abuse and psychological distress. The odds ratio of 2.44 suggests that older adults who experienced abuse during last month are more than twice as likely to report psychological distress than those with no such experience. This effect is

For peer review only - http://bmjopen.bmj.com/site/about/guidelines.xhtml

attenuated once psychosocial resources and other control variables are added (Model 2). Social activity participation, social support (having someone to trust) and mastery (feeling able to manage situations) are all associated with psychological distress in the expected direction. The exception is marital status, with the results indicating that older people who are currently married or living together with partners are more likely to have psychological distress than those who are widowed, although this finding is not statistically significant. Finally, Model 3 presents interactions between the experience of elder abuse and psychosocial factors (trust and mastery) and material resources (education and wealth quintile). The inclusion of interactions adds significant explanatory value to the model with a likelihood ratio test p-value of 0.008. The results indicate that psychosocial resources only have a direct association with psychological distress; the interaction terms between elder abuse and psychosocial resources variables are not statistically significant. Interestingly, however, positive and significant interactions are observed between the experience of elder abuse and the respondents' household wealth quintile.

Figure 2 shows the predicted probabilities for GHQ-12 ≥ 4 at each household wealth quintile according to elder abuse experience, based on the coefficients from Model 3. The chart shows that for those who did not experience elder abuse experience in the last month, the probability of psychological distress decreases with the increase of household wealth. However, amongst those who had experienced abuse, the opposite is found, with the probability of psychological distress increasing as household wealth rises.

For peer review only - http://bmjopen.bmj.com/site/about/guidelines.xhtml

Discussion and conclusion

The analyses in this paper suggest that elder abuse has a significant association with the mental health of older Indians. The results support the first hypothesis outlined in this paper. Elder abuse may be thought of as a particularly stressful event in later life. Typically Indian parents have continued to invest in their children into adulthood and traditionally have expected to be cared for at an older age. If their investment is not reciprocated, their life is likely to be coloured by a sense of injustice and exploitation, [12] which may lead to certain negative effects such as anger, depressed mood and loneliness. [34] Constant negative effects are known to be compromising to both physical and mental health, with the mechanism of pathogenesis operating through physiological changes, including one's immune suppression, and cardiovascular and endocrine reactivity. [15,35] Our results are consistent with other empirical studies, suggesting that there is a harmful link between older abuse and psychological health. [23,36,16,17] However, no evidence is found for buffering effects of psychosocial resources, such as social support and perceived ability to control outcomes. The results only highlight a direct and beneficial association between psychosocial resources and psychological health, irrespective of the presence of elder abuse. One possible explanation is that elder abuse is in direct conflict with Indian cultural values, and thus older adults who have been abused may not disclose this information or seek support due to a sense of shame and/or a fear of stigmatization. [37-38] Another explanation may lie in the scale of the outcome variable. In this study, we focus on psychological distress using a nominal scale measurement. Previous studies that have demonstrated the buffering effects of psychosocial resources have measured psychological distress as a ratio scale [23,17] and thus it is possible that by using a

For peer review only - http://bmjopen.bmj.com/site/about/guidelines.xhtml

nominal scale we may be missing some of the nuances around buffering effects. Interestingly the results in this paper show that household wealth has a direct and inverse relationship with psychological distress, and also offers a substantial link with the relationship between elder abuse and psychological distress. Both qualitative and quantitative studies have found that individuals who have financial or physical assets may feel more in control of their lives, leading to less vulnerability to anxiety or mood disorders, or less severe psychological symptoms. [8,39] Unexpectedly, however, we found that the negative association between elder abuse and mental health is significantly stronger among older people living in wealthier households. One possible explanation might be that issues of control over property, finance, and other decisions may result in more family conflict between parents and their adult children or other relatives among wealthier households than in poorer households. This is consistent with qualitative studies in India which have highlighted bitter battles in village families between elders and adult children over land and money. [8,6] Adult children have been reported at times to resent the expense of medical care and treatments for their aged parents, especially when some of the children felt they were providing more than their fair share of the total cost. Again this may be more commonplace amongst wealthier households, where private medical care is an option. Our findings reinforce previous research demonstrating the role of socio-economic circumstances in determining older people's mental health. Poor social and economic circumstances affect individuals' health throughout life. [40-42] The results also add to the evidence base with regard to inequalities in older people's mental health related to levels of physical health [43-46] and different geographic areas, reflecting differences

For peer review only - http://bmjopen.bmj.com/site/about/guidelines.xhtml

in their social, political, economic arrangements and levels of public health services and social protection.[47]

In conclusion, the findings presented in this paper demonstrate that 1) in the seven Indian states represented in this research, elder abuse shows a negative association with the mental health of older adults; 2) household wealth generally has an inverse relationship with mental health; 3) however, the negative association between elder abuse and mental health is stronger among older people living in wealthy households.

The present study is limited by the cross-sectional design of the data. It is possible that older people already had stress before the experience of abuse, and at the time of the interview were more likely than others to recall past experiences such as abuse. The results could benefit from repeated measures of psychological distress before and after abusive exposures. Another limitation is the self-report nature of the data. There is no validation by agencies charged with investigating elder abuse. Due to social taboo, elder abuse might be underreported, which may bias our results on the relationship between abuse and distress. Our data also lack purposively designed scales measuring social support and personal coping resources. Future research addressing these issues will improve our understanding of the relationship between elder abuse, psychosocial resources, and psychological distress.

The number of older people in India is steadily growing. [4] Increased life expectancy brings with it more chronic health problems and functional limitations that require long-term care. Most older people continue to live in villages and to experience poor socioeconomic status and are dependent upon their families for both financial and physical support. While the need

For peer review only - http://bmjopen.bmj.com/site/about/guidelines.xhtml
For peer review only

for care has grown, available resources have decreased. The lack of jobs close to where they live forces many young Indians to seek employment in urban areas. Such migration reduces the number of available caregivers and increases the demands on non-migrant family members to shoulder responsibility for elders' care. The costs of care were often high, due to a lack of adequate public health care for older persons. Mistreatment of one's older parents may also emanate from conflict over the control of family property. Researchers have begun to argue that traditional Indian cultural values and the consequences of urbanization and modernization influence the nature and scope of elder abuse. [38] In addition, recent research has highlighted the increasing incidence of elder abuse due to property separation/division, living conditions and the growing generation differences in thinking and attitude towards expectations and lifestyles. [48] As India continues on its path of economic development, with increasing urbanisation and spatial mobility, older people may be further exposed to abuse.

At present, mental health in later life is not a priority area in many low income countries [49] and how it is associated with elder abuse is neglected in both the research and policy arenas. Intergenerational relations between older people and their adult children are pivotal in the health and wellbeing of older people. However, such relations can have both positive and negative impacts upon the mental wellbeing of both the older person and, in certain cases, that of the adult child carer. [50-51] Elder abuse needs to be recognised as a key public health issue, and appropriate strategies, policies and practices put in place. Reducing elder abuse will have a positive impact on both the physical and mental health outcomes in later life. Policy makers in India are faced with a major challenge in a low resource context, however

For peer review only - http://bmjopen.bmj.com/site/about/guidelines.xhtml

widening the public policy debate to include the recognition of the prevalence of elder abuse and how best to address it within health policy planning would be a key move forward.

Contributors All authors Maria Evandrou, Jane Falkingham, Min Qin and Athina Vlachantoni contributed to conceptualising, writing, editing this paper, and have read and approved the final manuscript. Data analysis was performed by Min Qin.

Funding The study was supported under the NWO-ESRC-ICSSR AgeGlobe Network (Indian-European research networking: ageing and well-being in a globalising world; grant number ES/K005979/1).

Competing interests None declared.

Ethics approval Ethical approval for this study has been obtained from the Ethics Committee in the University of Southampton. The ethics clearance for the original study was obtained by the UNFPA.

Data sharing statement No additional data are available.

For peer review only

For peer review only - http://bmjopen.bmj.com/site/about/guidelines.xhtml

References

1 United Nations (UN). *World Population Prospects 2015*. New York: UN Department of Economic & Social Affairs, Population Division, 2015.

2 Dhillon P, Ladusingh L, Agrawal G, Ageing and changing patterns in familial structure for older persons in India: a decomposition analysis. *Quality in Ageing and Older Adults* 2016; 17: 83 – 96. doi: 10.1108/QAOA-10-2014-0024

3 Venkoba RA. Psychiatry of old age in India. *International Review of Psychiatry* 1993; 5: 165–170.

4 Government of India. *Situation Analysis of the Elderly in India*. Central Statistics Office, Ministry of Statistics & Programme Implementation, 2011. http://mospi.nic.in/mospi_new/upload/elderly_in_india.pdf (accessed 14 Oct 2016)

5 Patel V, Prince M. Ageing and mental health in developing countries: who cares? Qualitative studies from Goa, India. *Psychological Medicine* 2001; 31: 29–38. PMID: 11200957

6 Srivastava S, Bhatia MS, Rajoura OP, *et al.* Elder neglect in changing Indian scenario. *Delhi Psychiatry Journal* 2013; 16: 273–276. http://medind.nic.in/daa/t13/i2/daat13i2p273.pdf(accessed 17 Oct 2016)

7 WHO. *A Global Response to Elder Abuse and Neglect: Building Primary Health Care Capacity to Deal with the Problem Worldwide: Main Report* (pp.1). Geneva: WHO, 2008.

8 Soneja S. *Elder Abuse in India* Country Report for World Health Organization.1999. http://www.who.int/ageing/projects/elder_abuse/alc_ea_ind.pdf (accessed 17 Oct

For peer review only

2016)

9 Skirbekk V, James KS. Abuse against elderly in India – the role of education. *BMC Public Health* 2014; 14: 336. http://www.biomedcentral.com/1471-2458/14/336. (accessed 14 Oct 2016)

10 Ohayashi H, Yamada S. *Psychological Distress Symptoms, Causes and Coping*. New York: Nova Science Publishers, Inc. 2012

11 Tyrer P, Steinberg D. *Models for Mental Disorder*. Chichester: John Wiley and Sons. 2013.

12 Winterstein T, Eisikovits Z. The experience of loneliness of battered old women. *Journal of Women & Aging* 2005; 17: 3–19. doi:10.1300/J074v17n04_02

13 Dyer CB, Pavlik VN, Murphy KP, *et al.* The high prevalence of depression and dementia in elder abuse or neglect. *JAGS* 2000; 48: 205-208. PMID: 10682951

14 Begle AM, Strachan M, Cisler JM, *et al.* Elder mistreatment and emotional symptoms among older adults in a largely rural population: the South Carolina elder mistreatment study. *Journal of Interpersonal Violence* 2011; 26: 2321–2332. doi: 10.1177/0886260510383037

15 Selye H. Stress and disease. *Science*1955; 122: 625–631. doi: 10.1126/science.122.3171.625

16 Dong XQ, Simon MA, Beck TT, *et al.* Elder abuse and mortality: the role of psychological and social wellbeing. *Gerontology* 2011; 57: 549–558. doi: 10.1159/000321881

17 Luo Y, Waite LJ, Mistreatment and psychological well-being among older adults:

For peer review only

For peer review only - http://bmjopen.bmj.com/site/about/guidelines.xhtml

exploring the role of psychosocial resources and deficits. *J Gerontol B Psychol Sci Soc Sci,* 2011; 66: 217–229. doi: 10.1093/geronb/gbq096

18 Fisher BS, Regan SL, The extent and frequency of abuse in the lives of older women and their relationship with health outcomes. *Gerontologist* 2006; 46: 200–209. doi: 10.1093/geront/46.2.200

19 Cohen S, Wills TA. Stress, social support, and the buffering hypothesis. *Psychological Bulletin* 1985; 98: 310–357.

20 Lazarus R S, Folkman S, *Stress, Appraisal and Coping*. New York: Springer, 1984.

21 Pearlin LI, Schieman S, Fazio EM, *et al.* Stress, health, and the life course: Some conceptual perspectives. *Journal of Health and Social Behavior* 2005; 46: 205–219. doi:10.1177/002214650504600206

22 Taylor SE, Stanton AL. Coping resources, coping processes, and mental health. *The Annual Review of Clinical Psychology* 2007; 3: 377–401. doi:10.1146/annurev.clinpsy.3.022806.091520

23 Comijs HC, Penninx BW, Knipscheer, KP, *et al.* Psychological distress in victims of elder mistreatment: the effects of social support and coping. *J Gerontol B Psychol Sci Soc Sci,* 1999; 54: 240–245. PMID: 12382593

24 United Nations Population Fund (UNFPA). *Report on the status of elderly in select states of India, 2011*. New Delhi: United Nations Population Fund, 2012.

25 Goldberg DP, Gater R, Sartorius N, *et al.* The validity of two versions of the GHQ in the WHO study of mental illness in general health care. *Psychological Medicine* 1997; 27: 191–197. doi: 10.1017/S0033291796004242

For peer review only - http://bmjopen.bmj.com/site/about/guidelines.xhtml

26 Shamasunder C, Srram TG, Murali RSG, *et al.* Validity of a short 5 - item version of the General Health Questionnaire (GHQ). *Indian J Psychiatry* 1986; 28: 217–219. PMCID: PMC3172533

27 Shivakumar P, Sadanand S, Bharath S, *et al.* Identifying psychological distress in elderly seeking health care. *Indian J Public Health* 2015; 59: 18–23. doi:10.4103/0019-557X.152849

28 Gautam S, Nijhawan M, Kamal P, Standardisation of Hindi version of Goldberg's General Health Questionnaire. *Indian J Psychiatry* 1987; 29: 63-66. PMID: 21927210

29 Kuruvilla A, Pothen M, Philip K, *et al.* The validation of the Tamil version of the 12 item general health questionnaire. *Indian J Psychiatry* 1999; 41: 217–221. PMCID: PMC2962995

30 Goldberg D, Oldehinkel T, Ormel J, Why GHQ threshold varies from one place to another. *Psychological Medicine* 1998; 28: 915–921. PMID: 9723146

31 Pearlin LI, Schooler C. The structure of coping. *Journal of Health and Social Behavior* 1978; 22, 337–356. PMID: 649936

32 Sherbourne CD, Stewart AL. The MOS social support survey. *Social Science & Medicine* 1991; 32: 713–714. PMID: 2035047

33 Timmerman IGH, Emanuels-Zuurveen ES, Emmelkamp PMG. The Social Support Inventory (SSI): A Brief Scale to Assess Perceived Adequacy of Social Support. *Clin Psychol Psychother* 2000; 7: 401–410. doi:10.1002/1099-0879(200011)7:5<401::AID-CPP253>3.0.CO;2-I

34 Martin P, Hagberg B, Poon LW. Predictors of loneliness in centenarians: A parallel study.

For peer review only

For peer review only - http://bmjopen.bmj.com/site/about/guidelines.xhtml

J Cross Cult Gerontol 1997; 12: 203–224. PMID: 14617927

35 Tachè J, Selye H. On stress and coping mechanisms. *Issues in Mental Health Nursing* 1985; 7: 1–4, 3–24. doi: 10.3109/01612848509009447

36 Chokkanathan, S. Resources, stressors and psychological distress among older adults in Chennai, India. *Social Science & Medicine* 2009; 68: 243–250. doi: 10.1016/j.socscimed.2008.10.008

37 Segal U. Family violence: a focus on India. *Aggression and Violent Behavior* 1999; 4: 213–231. doi:10.1016/S1359-1789(97)00051-7

38 Nagpaul K. Elder abuse among Asian Indians: traditional versus modern perspectives. *Journal of Elder Abuse & Neglect* 1998; 9: 77–92. doi: 10.1300/J084v09n02_07

39 Carter KN, Blakely T, Collings S, *et al.* What is the association between wealth and mental health? *J Epidemiol Community Health* 2008; 63: 221–226. doi:10.1136/jech..079483

40 WHO. *The Solid Facts: Social Determinants of Health*. Copenhagen: World Health Organization Regional Office for Europe, 2003.

41 Gallo LC, Matthews KA, Understanding the association between socioeconomic status and physical health: do negative emotions play a role? *Psychological Bulletin* 2003; 129: 10–51. doi: 10.1037/0033-2909.129.1.10

42 Jeon GS, Jang SN, Rhee SJ, *et al.* Gender differences in correlates of mental health among elderly Koreans. *J Gerontol B Psychol Sci Soc Sci,* 2007; 62B, 5, S323–S329. PMID: 17906176

43 Ormel J, Kempen GIJM, Penninx BWJH, *et al.* Chronic medical conditions and mental

For peer review only - http://bmjopen.bmj.com/site/about/guidelines.xhtml

health in older people: disability and psychosocial resources mediate specific mental health effects. *Psychological Medicine* 1997; 27: 1065 –1077. PMID: 9300512

44 Joshi K, Kumar R, Avasthi A, Morbidity profile and its relationship with disability and psychological distress among elderly people in Northern India. *Int J Epidemiol* 2003; 32: 978–987. PMID: 14681260

45 Shih M, Simon PA. Health-related quality of life among adults with serious psychological distress and chronic medical conditions. *Qual Life Res* 2008; 17, 521–528. doi:10.1007/s11136-008-9330-9

46 Rajkumar AP, Thangadurai P, Senthilkumar P, *et al.* Nature, prevalence and factors associated with depression among the elderly in a rural South Indian community. *International Psychogeriatrics* 2009; 21: 372–378. doi: 10.1017/S1041610209008527

47 World Health Organization, Calouste Gulbenkian Foundation (WHO, CGF). *Social Determinants of Mental Health*. Geneva: World Health Organization, 2014.

48 Kumar Y, Bhargava A, Elder abuse in Indian families: problems and preventive actions. *IJSRP* 2014; 4: 1–8. http://www.ijsrp.org/research-paper-1014/ijsrp-p3498.pdf (accessed 14 Oct 2016)

49 Patel V. Mental health in low- and middle-income countries. *British Medical Bulletin* 2007; 81 and 82, 81–96. doi: 10.1093/bmb/ldm010

50 Stone J, Evandrou M, Falkingham J. The transition to living alone and psychological distress in later life. *Age and Ageing* 2013; 42: 366–372. doi:10.1093/ageing/aft006.

51 Vlachantoni A, Robards J, Falkingham J *et al.* Trajectories of informal care and health. *SSM -Population Health* 2016; 2: 495–501. doi:10.1016/j.ssmph.2016.05.009

For peer review only

Table 1. Distribution of GHQ-12 ≥ 4 (unweighted data)

Variables	Distribution (%)	Number of respondents	% of GHQ-12 score ≥ 4	P value (Pearson Chi square test)
**Total**	100.0	9,589	40.6	
Experience abuse last month				
No	95.5	9,157	39.7	.000
Yes	4.5	432	61.6	
**Selected psychosocial and material resources variables**				
Marital status				
Widowed	40.5	5,710	48.0	.000
Currently married/living together	59.5	3,879	35.7	
Social activities				
No listed social activity	19.4	1,860	55.8	.000
Have social activity	80.6	7,729	37.0	
Have someone trust or confide				
No	17.1	1,642	59.7	.000
Yes	82.9	7,947	36.7	
Feel able to manage situations				
Hardly ever	24.0	2,298	76.3	.000
Sometimes	63.2	6,057	32.8	
Most of the time	12.9	1,234	12.9	
Income				
No income	47.5	4,553	46.2	
One source	44.7	4,290	36.9	
Multiple sources	7.8	746	28.3	
Household wealth index				
Lowest	20.0	1,915	64.1	.000
Second	20.4	1,958	48.4	
Middle	19.6	1,884	40.4	
Fourth	19.8	1,901	28.8	
Highest	20.1	1,931	21.4	
**Other control variables**				
age				
60-69	63.4	6,082	35.6	.000
70-79	26.4	2,533	46.9	
80+	10.2	974	55.9	
Sex				
Men	47.4	4,543	36.2	.000
Women	52.6	5,046	44.7	
Education				
None	46.1	4,422	52.1	.000
1-4 years	12.9	1,241	45.2	
5-7 years	13.5	1,297	36.9	
8+ years	27.4	2,629	21.0	
Caste				
Scheduled Tribe/Scheduled Caste	24.2	2,316	47.4	.000
Other Backward Caste	34.1	3,274	42.9	
Others	39.1	3,753	33.4	
Unknown	2.6	246	58.5	
Working status				
Has never worked	36.2	3,472	41.2	.000
Has ever worked but not now	40.7	3,904	42.6	
Has ever worked and is now working	23.1	2,213	36.2	
Self-reported health				
Excellent /Very good	16.2	1,557	19.1	.000
Good	30.0	2,875	28.1	
Fair	36.5	3,499	48.7	
Poor	17.3	1,658	65.6	
Chronic disease				
No	35.3	3,389	35.3	.000
1 type	32.2	3,087	41.7	
2 more types	32.5	3,113	45.4	
ADL				
No need for assistance	92.7	8,890	38.1	.000
Light need	3.8	369	67.8	
Heavy need	3.4	330	79.1	
Disability				
No disability	27.2	2,606	25.1	.000
Light	44.7	4,285	37.6	

For peer review only

Variables	Distribution (%)	Number of respondents	% of GHQ-12 score ≥ 4	P value (Pearson Chi square test)
Medium	18.6	1,788	54.5	
Heavy	9.5	910	72.0	
Living arrangement				
Alone	6.3	605	50.9	.000
Spouse only	14.9	1,432	34.9	
At least one child	71.3	6,833	40.5	
Others	7.5	719	44.6	
Residence				
Rural	52.2	5,001	45.7	.000
Urban	47.8	4,588	35.1	
State				
Himachal Pradesh	15.0	1,440	23.5	.000
Punjab	13.1	1,255	20.8	
West Bengal	13.2	1,263	60.5	
Odisha	15.3	1,467	55.5	
Maharashtra	14.6	1,399	44.3	
Kerala	14.0	1,341	28.0	
Tamil Nadu	14.9	1,424	50.8	

Source: Authors' analysis of UNFPA Building Knowledge Base on Ageing in India 2011 survey

For peer review only

For peer review only - http://bmjopen.bmj.com/site/about/guidelines.xhtml

Table 2.Odds ratios for GHQ-12 score ≥ 4(N=9,589)

Variables	Model 1	Model 2	Model 3
Experienced abuse in last month No (ref)			
Yes	2.44***	1.60***	1.11
**Psychosocial and material resources**			
Marital status Widowed (ref)			
Currently married/living together		1.14	1.14
Social activities participation No (ref)			
Yes		0.85*	0.85*
Have someone trust or confide No (ref)			
Yes		0.69***	0.69***
Feel able to manage situations Hardly ever (ref)			
Sometimes		0.25***	0.25***
Most of the time		0.12***	0.12***
Income No income (ref)			
One resources		0.97	0.96
Multiple resources		0.74*	0.73**
Household wealth index Lowest (ref)			
Second		0.83*	0.80*
Middle		0.78**	0.73***
Fourth		0.62***	0.57***
Highest		0.53***	0.49***
**Other control variables**			
Age 60-69 (ref)			
70-79		1.20**	1.21**
80+		1.23*	1.25*
Gender Men (ref)			
Women		1.15	1.17
Education No schooling (ref)			
1-4 years		0.88	0.88
5-7 years		0.85	0.85
8+ years		0.56***	0.57***
Caste **Scheduled Tribe/Scheduled Caste (ref)**			
Other Backward Caste		0.92	0.92
Others		1.05	1.07
Unknown		1.09	1.07
Working status Not work (ref)			
Ever work but not now		1.19*	1.23*
Ever work and now		1.08	1.12
Self-reported health Excellent/very good/good (ref)			
Fair		2.11***	2.11***
Poor		3.88***	3.90***
Chronic disease No (ref)			
One type		1.17*	1.17*
More than one types		1.31***	1.31***
Difficulty with ADLs No need for assistance (ref)			
Light need		1.21	1.23
Heavy need		1.82***	1.83***
Disability No disability (ref)			
Light		1.56***	1.56***
Medium		2.24***	2.22***
Heavy		3.40***	3.38***
Living arrangement Alone (ref)			
Spouse only		0.80	0.80
At least one child		1.16	1.16
Others		1.25	1.25
Residence Rural (ref)			
Urban		0.90	0.90
State Himachal Pradesh (ref)			
Punjab		0.59***	0.60***
West Bengal		2.70***	2.66***
Odisha		2.85***	2.81***
Maharashtra		1.71***	1.68***
Kerala		0.98	0.98
Tamil Nadu		3.89***	3.82***
Abuse* Second quintile			1.39
Abuse * Middle quintile			2.96**

For peer review only

Variables	Model 1	Model 2	Model 3
Abuse * Fourth quintile			4.37***
Abuse * Highest quintile			4.57**
Abuse * High education			0.74
Abuse * Have someone to trust			0.87
Abuse * Feel able to manage situations some time			0.92
Abuse * Feel able to manage situations most time			1.02
**Constant**	0.66***	0.70	0.72
**Cox & Snell R^2**	0.008	0.327	0.328
**Nagelkerke R^2**	0.011	0.441	0.442

Source: Authors' analysis of UNFPA Building Knowledge Base on Ageing in India 2011 survey

For peer review only

Figure 1. Predicted probability of GHQ-12>=4 among older adults by elder abuse and household wealth quintile

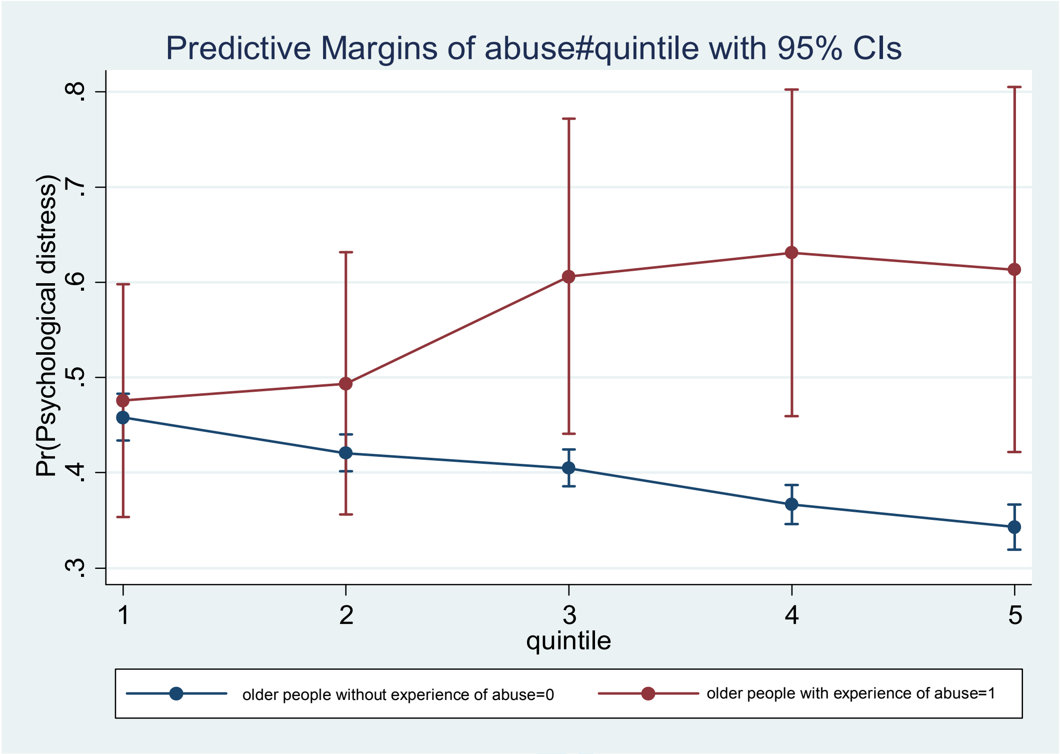

Source: Authors' analysis of UNFPA Building Knowledge Base on Ageing in India 2011 survey

For peer review only - http://bmjopen.bmj.com/site/about/guidelines.xhtml

BMJ Open

Elder abuse as a risk factor for psychological distress among older adults in India: a cross-sectional study

Journal:	*BMJ Open*
Manuscript ID	bmjopen-2017-017152.R1
Article Type:	Research
Date Submitted by the Author:	21-Aug-2017
Complete List of Authors:	Evandrou , Maria ; University of Southampton, Centre for Research on Ageing, ESRC Centre for Population Change<br>Falkingham, J; University of Southampton<br>Qin, Min; Centre for Research on Ageing, ESRC Centre for Population Change<br>Vlachantoni, Athina; University of Southampton
**Primary Subject Heading**:	Public health
Secondary Subject Heading:	Mental health
Keywords:	Elder abuse, Risk factors, MENTAL HEALTH, Psychological distress, India

SCHOLARONE™
Manuscripts

For peer review only

Elder abuse as a risk factor for psychological distress among older adults in India: a cross-sectional study

Maria Evandrou[1], Jane Falkingham[1], Min Qin[1] and Athina Vlachantoni[1]

[1]Centre for Research on Ageing and ESRC Centre for Population Change, University of Southampton, Southampton, SO17 1BJ, UK

Correspondence to

Dr. Min Qin

Centre for Research on Ageing and ESRC Centre for Population Change

University of Southampton

Southampton, SO17 1BJ

UK

Tel +44 (0)23 80 594290

Fax +44 (0)23 80 594290

Email: min.qin@soton.ac.uk

Word count 5014

For peer review only

For peer review only - http://bmjopen.bmj.com/site/about/guidelines.xhtml

Abstract

Objectives This study examines the association between elder abuse and psychological distress among older adults in India, and explores whether this association varies by the level of psychosocial and material resources.

Design The study uses a cross-sectional survey design.

Setting The data are drawn from a representative sample of 9,589 adults aged 60 and above in seven Indian states - Himachal Pradesh, Punjab, West Bengal, Odisha, Maharashtra, Kerala and Tamil Nadu – in 2011.

Statistical analyses Secondary analysis, using bi-variate and multivariate logistic regression models, is conducted using the UNFPA project Building Knowledge Base on Ageing in India (BKPAI) survey. Elder abuse (physical and/or emotional) emanating from family members in the previous month before the survey is examined. Multivariate models are run on the total analytical sample, and for men and women separately.

Results The overall prevalence of psychological distress amongst persons aged 60 and over living in the seven Indian States is 40.6 percent. Among those older persons who experienced some form of physical or emotional abuse or violence in the last month, the prevalence of psychological distress is much higher than that in the general older population, at 61.6 percent ($P<0.001$). The results show that the experience of abuse is negatively associated with the mental health of older adults, and this relationship persists even after controlling for demographic and socio-economic factors (OR=1.60, 95% CI=1.22-2.09). The findings also suggest that household wealth has an inverse relationship with mental health, with the

For peer review only - http://bmjopen.bmj.com/site/about/guidelines.xhtml

association between experiencing elder abuse and reporting poor mental health being strongest amongst older people in wealthy households.

Conclusions Elder abuse in India is currently a neglected phenomenon, and greater recognition of the link between abuse and mental health is critical in order to improve the well-being of vulnerable older adults, some of whom may be 'hidden' within well-off households.

Key Words: Elder abuse—Risk factors—Mental health—Psychological distress—India

Strengths and limitations of this study

- The findings of the study are from a large representative sample of 9,692 older adults from selected Indian states.
- Abuse data in this study are self-reported; there is no validation (or under-reporting) by agencies charged with investigating elder abuse.
- It is possible that older people already had stress before the experience of abuse, and at the time of the interview were more likely than others to recall past experiences such as abuse.

For peer review only - http://bmjopen.bmj.com/site/about/guidelines.xhtml

INTRODUCTION

India's population is ageing. With improvements in mortality as a result of rising living standards, improved sanitation, public health and medical advances, more people are living longer and surviving into old age. Such trends, combined with recent falls in fertility, mean that the number of older people is both increasing in absolute terms and also as a share of the population. In 1980, individuals aged 60 and over accounted for just 5.9 percent of the Indian population; by 2015 this had risen to 8.9 percent, comprising 116.5 million people, and by 2050 older people are projected to constitute nearly one fifth (19.4%) of the total population, with 330 million Indians in their sixties or older. [1] The changes in the age structure of India's population are being accompanied by other social and economic transformations including rapid urbanization and industrialization. Increasing women's participation in paid employment, greater internal and international mobility amongst the younger generation and the growth of individualism are all impacting upon the traditional Indian family system; a system which has emphasized the obligation of sons and their wives to respect, obey, and provide care for their aged parents. [2]

Traditionally, elders have been respected in Indian society, and families are the principal financial, emotional and physical caregivers for older relatives. [3] Although this tradition remains today, [4] qualitative studies have demonstrated that both respect for older people and the caring traditions of the extended family are on the wane in the larger context of societal and cultural changes. Older people are more likely to be exposed to abuse, isolation and abandonment. [5-6] Elder abuse is internationally defined as a "single, or repeated act, or lack of appropriate action, occurring within any relationship where there is an expectation of trust

For peer review only - http://bmjopen.bmj.com/site/about/guidelines.xhtml

which causes harm or distress to an older person". [7] Elder abuse is estimated to affect one in six older adults worldwide, has become a growing public health challenge and requires more attention by health care systems, researchers, and more evidence based intervention. [8-9] Across a wide range of countries, risk factors for elder abuse include functional dependence or physical disability, poor physical and mental health and low socio-economic status. Most international studies found women are more likely than men to experience elder abuse. [10]

Several theories may explain the possible causes of elder abuse by family members. [11] According to the social exchange theory, elder abuse may arise because of older people's dependence on the family members, while situational theory focuses on the role of stress and the burden of caregiving as precursors to elder abuse. An overburdened family member who cannot cope with caring demands may create an environment which is conducive to abuse. Symbolic interactionism theory emphasizes the role of cultural values and expectations in influencing the perception of elder abuse. For example, in some elders cultural perceptions, going to live in a nursing homes is considered to be a form of abuse, whereas their children may define it as a sign of caring.

Elder abuse can manifest as physical, emotional, sexual, and financial abuse, and/or as intentional or unintentional neglect. In the Indian context, older people customarily perceive the word 'abuse' to mean extreme behaviour of violence, but not neglect/abandonment. However, in qualitative studies, older people have acknowledged the existence of maltreatment (lack of dignified living and disrespect) and neglect within their society and community. In addition, women have been considered as the worst sufferers with no income of their own and being dependent on other family members for everything. [12] Qualitative

For peer review only - http://bmjopen.bmj.com/site/about/guidelines.xhtml

research has found that selected later life mental disorders may be attributed to abuse, neglect, or a lack of love from children. [5] However, psychological distress in later life remains an under-researched area in India, particularly in terms of the psychological consequences associated with elder abuse and neglect. [12-13]

THE RELATIONSHIP BETWEEN ELDER ABUSE, RESOURCES AND PSYCHOLOGICAL DISTRESS

Psychological distress is widely used as an indicator of the mental health of the population within the field of public health (psychological distress and poor mental health are interchangeable terms in this paper). Distress comprises of a variety of symptoms such as depression, anxiety, stress and insomnia. The level of such distress experienced by an individual at any point in time is determined by various biological and psychosocial factors. [14-15] Elder abuse is recognised as a stressful experience which has been found to have harmful effects on mental health, [16] with depression, anxiety and post-traumatic disorder being reported as the most prevalent psychological consequences. [17-18]

The general stress theory postulates that the effect of stressors (i.e. stressful events) on psychological health operates in three phases: alarm, resistance, and exhaustion, and is a process that involves changes in individuals' immune system, endocrine system and cardiovascular reactivity. [19] When problems accumulate, persist and strain individuals, then adaptation resources are depleted and a stimulated parasympathetic system may lead to worrying, anxiety, depression, anger and/or other physical illness. Studies have found that

For peer review only - http://bmjopen.bmj.com/site/about/guidelines.xhtml

older adults who are mistreated have higher levels of psychological distress than those who have no such experience. [20-21] The frequency or type of elder abuse also has an impact on mental health. Fisher and Regan (2006) [22] found that repeated abuse or multiple types of elder abuse (e.g. emotional) were risk factors for depression or anxiety among older women. Based on previous empirical findings, this paper hypothesizes (H1) *that older adults who report experiencing elder abuse will have higher odds of psychological distress than those who do not report such experiences*.

However, many individuals who experience stressful events do not go on to develop a psychological illness. The stress-buffering model suggests that psychosocial resources moderate the deleterious effects of high levels of stress. The statistical interaction between stress and resources can be used to test these moderating effects. [23] Resources may intervene between the experience of stress and the onset of mental illness by providing a solution to the problem or reducing the perceived importance of the problem, which in turn helps to decrease or eliminate the stress reaction. Psychosocial resources that can buffer the negative impact of life events on psychological well-being include subjective resources such as high self-esteem, mastery, social support and social participation, and objective resources such as socioeconomic status, including income and household wealth.[24-26] For instance, a beneficial effect of social support on one's mental health could occur thanks to large social networks providing individuals with regular positive experiences and stable, socially-rewarding roles in the community. This kind of support could provide a positive effect, a sense of predictability and stability in one's life situation, and recognition of self-worth, all of which are related to overall well-being. At the same time, material resources such as income and household

For peer review only - http://bmjopen.bmj.com/site/about/guidelines.xhtml

wealth can offer protection against negative experiences associated with economic problems. Indeed, previous empirical studies have found that in the presence of stress from elder abuse, supportive relationships may buffer the effect of stress. [27, 21] As such, this paper hypothesizes that (H2) *the negative association of elder abuse with psychological distress will be stronger for those with fewer psychosocial and material resources than for those with more psychosocial and material resources*. This study aims to contribute to the literature by investigating the association between elder abuse and psychological distress among older adults in India and examining whether such association varies by the level of psychosocial and material resources at the older adults' disposal.

DATA AND METHODS

This study analyses data collected as part of the UNFPA 'Building Knowledge Base on Ageing in India (BKPAI)' project. The BKPAI Survey was conducted in 2011 in seven major demographically advanced states of India - Himachal Pradesh, Punjab, West Bengal, Odisha, Maharashtra, Kerala and Tamil Nadu. A representative sample was obtained using a random sampling method covering the Northern, Southern, Western and Eastern regions. The detailed information about the survey sampling is described in a previous report. [28] The primary sampling units were households. All those aged 60 and above in the sampled households were interviewed face-to-face. The completion rate for households was 94.7 percent and 92.9 percent for elderly respondents. Non-response at both the household and individual levels was adjusted through the sampling weights calculation by the research organisation. The

For peer review only - http://bmjopen.bmj.com/site/about/guidelines.xhtml

For peer review only

BKPAI survey data includes information on older people's mental and physical health, their living arrangements, socio-economic circumstances, including employment status and household assets, as well as information on intergenerational exchanges within the family and participation in social activities. The total sample size interviewed is 9,692. Of these, 103 are excluded because of missing values (missingness is not mutually exclusive) on psychological distress (N=36); education (N=53); whether has someone for trust/confidence (N=7); and whether feels able to manage unexpected situations (N=16). The final analytical sample is 9,589 adults aged 60 and above.

Measurements

Elder abuse

In the BKPAI survey, the respondents were asked two sets of questions regarding their experience of abuse since they were 60 years old and in the last month. The first question was *'In the time since you completed 60 years of age have you faced any type of abuse or violence or neglect or disrespect by any person?'* If the respondent answered 'Yes', a follow-up questions asked the type of abuse (Physical abuse, Verbal abuse, Economic abuse, Showing disrespect, Neglect and Other) and where it originated (within family, outside family, both within family & outside family). *A further question asked* '*Have you faced any type of physical or emotional abuse or violence in the last month?'* The responses include '*1. No, 2. Physical, 3. Emotional, 4. Both physical and emotional*'. All other types of violence other than physical were merged into emotional violence. If the respondent answered in the

For peer review only - http://bmjopen.bmj.com/site/about/guidelines.xhtml

For peer review only

affirmative, follow-up questions elicited the source of abuse which could include '*1. Spouse, 2. Son, 3. Daughter, 4. Son-in-law, 5. Daughter-in-law, 6. Domestic helper, 7. Grandchildren, 8. Relatives, 9. Neighbours, 10. Other*'.

A previous study based on this data reported that 11 percent of respondents have experienced at least one type of abuse *after the age of 60*. Verbal abuse is most frequently claimed, followed by disrespect, economic abuse, neglect and physical abuse, with the most common perpetrator being the respondent's son. [17] In this study we concentrate on older adults who report having experienced physical and/or emotional abuse in *the last month*, distinguishing between those who report abuse by family members and others, to examine the contemporaneous interaction between elder abuse, psychosocial and material resources, and psychological distress. Here, abuse is limited to that reported as emanating from family members as it is this form of abuse that we hypothesise may have increased as a result of recent changes impacting the traditional Indian family system.

Psychological distress

The 12-item version of the General Health Questionnaire (GHQ-12) is used as a measure of psychological distress. These questions have been widely used to identify minor psychiatric disorders in the general population. [29] The GHQ-12 has been previously validated in India in clinical surveys conducted in Kannada, [30-31] Hindi [32] and Tamil. [33] Given that the BKPAI study was conducted across multiple states with different languages and focused amongst older adults, it was important for the team to test the reliability of GHQ-12 within the BKPAI.

For peer review only - http://bmjopen.bmj.com/site/about/guidelines.xhtml

The measure was found to have high internal consistency with an overall Cronbach's alpha of 0.9. Examining each state individually, Cronbach's alpha ranged from a low of 0.7 in West Bengal to a high of 0.94 in Himachal Pradesh, suggesting that the measure may be considered to be valid across all seven states and in all the languages used.

Using the standard GHQ scoring method, the four category responses for each of the 12 questions are coded (0, 0, 1, 1), with the points summed to produce a total score ranging from 0-12. We used a score of >=4 as the threshold to define psychological distress according to studies validating the GHQ-12 against standardized psychiatric interviews. [34, 31] Although a Likert scale (0-1-2-3) scoring method is also widely used, a previous study found that for the GHQ-12, the GHQ scoring method was more effective than the Likert method when defining the distressed cases. [29]

Psychosocial and material resources

An individual's psychosocial resources include personal qualities such as optimism, psychological control or mastery, and self-esteem, as well as the availability of social support, all of which can help to manage stressful events and contribute to better health outcomes.[35,24,26] There are a variety of scales measuring social support and personal coping resources.[36-37] The BKPAI was not explicitly designed to measure psychosocial resources. It does, however, contain a number of important indicators of potential support and coping resources, including being married or living together with one's partner, participation in

For peer review only - http://bmjopen.bmj.com/site/about/guidelines.xhtml

social activities, having someone to trust and confide in, and feeling able to manage unexpected situations.

Participation in social activities is defined as having participated in any of the five listed activities in the last 12 months: attending a public meeting with discussion of local, community or political affairs; attending any group, club, society, union or organizational meeting; working with other people in your neighbourhood to fix or improve something; attending or participating in any religious programs/services (not including weddings and funerals); going out of the house to visit friends or relatives.

The question on *feeling able to manage unexpected situations* has three response categories: most of the time, sometimes, and hardly ever feeling that one can manage situations even when they do not turn out to be as expected.

Material resources include personal financial dependency (no dependency, partial dependency, full dependency) and household wealth quintile index. Household wealth quintile index is computed using principle component analysis (PCA) based on 30 assets and housing characteristics: household electrification; drinking water source; type of toilet facility; type of house; cooking fuel; house ownership; ownership of a bank or post-office account; and ownership of a mattress, pressure cooker, chair, cot/bed, table, electric fan, radio/transistor, black and white television, colour television, sewing machine, mobile telephone, any landline phone, computer, internet facility; refrigerator, watch or clock, bicycle, motorcycle or scooter, animal-drawn cart, car, water pump, thresher and tractor. This

For peer review only - http://bmjopen.bmj.com/site/about/guidelines.xhtml

For peer review only

measure was found to provide a good socio-economic gradient of health outcomes among older adults in the survey. [28]

Other control variables

Covariates include the individual's age group, sex, education, caste, working status, living arrangement, self-reported health, chronic disease, health-related limitations to daily activities, disability and geographic factors (rural/ urban residence, and state). Functionality is measured using two derived variables capturing a) an individual's reported need for assistance with Activities of Daily Living (ADL) and b) Disability. ADLs refer to the ability to perform basic daily activities; disability is associated with a decline of motor function. In general, a high score of disability represents a lower ability to perform ADLs. [38] ADL is computed based on the level of independence reported by the older person in carrying out the activities of feeding, bathing, dressing, toilet, mobility and continence. Each question has three response categories: 'Do not require assistance; Require partial assistance; Require full assistance'. These are scored as 0, 1 or 2 respectively and are then summed across the six questions, resulting in a total score ranging between 0-12. Given the unequal intervals between the score, rather treating it as a continuous variable, we group it into an ordered categorical variable. Older respondents are defined as having 'no need for assistance' if the total score is 0, as having a 'light need for assistance' if the total score is between 1-5, and as having a 'heavy need for assistance' if the total score is ≥ 6.

For peer review only - http://bmjopen.bmj.com/site/about/guidelines.xhtml

Disability is computed based on the respondents' level of reported ability to see, hear, walk, chew, speak and remember. Each question has three response categories 'Yes fully, Yes partially, No'. These are scored as 0, 1 or 2 respectively and are then summed across the six questions, resulting in a total score of 0-12. Again, because of the unequal intervals between the score, we group the total score into an ordered categorical variable. Older people are defined as having 'no disability' if the total score is 0, 'light disability' if the total score is between 1-2, 'medium disability' with a score of 3-4, and 'high disability' if the total score is ≥ 5.

Statistical Analyses

The bi-variate associations of psychological distress with exposures and potential risk factors are explored using the χ^2 test first. Then a series of logistic regression models are estimated with the dependent variable being the report of psychological distress (GHQ ≥ 4 contrast to GHQ ≤ 3). The first model estimates the bivariate association between elder abuse and psychological distress. The second model adds the measures of psychosocial and material resources and other control variables to estimate the association of elder abuse and psychosocial and material resources, with psychological distress after controlling for other covariates. The final model includes the interaction terms of elder abuse with psychosocial and material resources variables to the main-effects-only model to examine whether psychosocial and material resources buffer the association between elder abuse and

For peer review only - http://bmjopen.bmj.com/site/about/guidelines.xhtml

psychological distress. The logistic regression models are run for the total sample, and then separately for older men and women using the Statistic software STATA12.

Ethics approval

Ethical approval for this study, involving secondary data analyses, has been obtained from the Ethics Committee in the University of Southampton. The survey report and a previous study show that informed consent was obtained from all individuals prior to participation in the primary data collection exercise. Careful attention was paid to avoid the presence of any family members during the collection of data concerning elder abuse and to guarantee the anonymity of all participants and the confidentiality of information. [13, 28]

RESULTS

Descriptive findings

Table 1 presents descriptive statistics for the total analytical sample. The overall prevalence of psychological distress amongst persons aged 60 and over living in the seven Indian States is 40.6 percent. Around 5 percent of older adults had experienced some form of physical or emotional abuse or violence in the last month. Among this sub-group, the prevalence of psychological distress is much higher than in the general older population, at 61.6 percent ($P<0.001$).

The indicators of psychosocial resources and socio-economic status appear to have an inverse relationship with psychological distress, with those older people living in households in the highest (richest) wealth quintile having a prevalence rate of 21.4 percent compared to 64.1

For peer review only - http://bmjopen.bmj.com/site/about/guidelines.xhtml

percent amongst those living in households in the lowest (poorest) wealth quintile. Similarly, those who participated in social activities in the last month are less likely to experience psychological distress than those who did not (37 v. 55.8 percent respectively).

Indicators of health status including fair/poor self-related health, heavy difficulty with ADLs and disability all show a positive association with psychological distress. Older people living with their spouse only experience the lowest prevalence of psychological distress (34.9 percent), while those living alone show the highest prevalence (50.9 percent). Levels of psychological distress increase with age and are higher among older women (44.7 percent) than older men (36.2 percent). One's place of residence seems to play an important role with elders living in urban areas having a lower level of psychological distress than their rural counterparts (35.1 v. 45.7 percent). There are also considerable inter-state variations in the prevalence of psychological distress. A relatively low level of psychological distress is found among older adults in Punjab (20.8 percent) and Himachal Pradesh (23.5 percent), contrasting with much higher levels in West Bengal (60.5 percent) and Odisha (55.5 percent).

Multivariate analysis results

Table 2 shows the odds ratios from the logistic regression models. Among the total sample, Model 1 shows the simple bi-variate relationships between elder abuse and psychological distress. The odds ratio of 2.44 suggests that older adults who experienced abuse during last month are more than twice as likely to report psychological distress than those with no such experience. This effect is attenuated once psychosocial resources and other control variables

For peer review only - http://bmjopen.bmj.com/site/about/guidelines.xhtml

are added (OR=1.60, 95% CI=1.22-2.09) (Model 2). Social activity participation, social support (having someone to trust) and mastery (feeling able to manage situations) are all associated with psychological distress in the expected negative direction. The exception is marital status, with the results indicating that older people who are currently married or living together with partners are more likely to have psychological distress than those who are widowed, although this finding is not statistically significant. Household wealth has an inverse relationship with psychological distress. Older people's psychological distress is also related to the levels of physical health and different geographic areas. For instance, older people with poor self-rated health are more likely to have psychological distress than those with good health (OR=3.83, 95% CI=3.24-4.52); older people living in Tamil Nadu are more likely to have psychological distress than those living in Himachal Pradesh (OR=3.81, 95% CI=3.01-4.81). Finally, Model 3 presents interactions between the experience of elder abuse and psychosocial factors (trust and mastery) and material resources (education and wealth quintile). The inclusion of interactions adds significant explanatory value to the model with a likelihood ratio test p-value of 0.008. The results indicate that psychosocial resources only have a direct negative association with psychological distress; the interaction terms between elder abuse and psychosocial resources variables are not statistically significant. Interestingly, however, positive and significant interactions are observed between the experience of elder abuse and the respondents' household wealth quintile (OR=2.96, 4.37 and 4.57 for the abuse among middle, fourth and highest quintile).

The separate models by gender show similar patterns of bi-variate relationships between elder abuse and psychological distress (for men OR=2.14, 95% CI=1.58-2.90; for women,

For peer review only - http://bmjopen.bmj.com/site/about/guidelines.xhtml

OR=2.60, 95% CI=2.00-3.39). Interestingly among older men, the significant association disappear once psychosocial resources and other control variables are added, while among older women the association is attenuated but still significant (OR=1.76, 95% CI=1.23-2.50). The results of interactions between the experience of elder abuse and psychosocial factors and material resources among women show similar patterns with the total sample.

Figure 1 shows the predicted probabilities for GHQ-12 ≥ 4 at each household wealth quintile according to elder abuse experience, based on the coefficients from Model 3 among the total sample. The chart shows that for those who did not experience elder abuse in the last month, the probability of psychological distress decreases with the increase of household wealth. However, amongst those who had experienced abuse, the opposite is found, with the probability of psychological distress increasing as household wealth rises.

DISCUSSION

The analyses in this paper suggest that elder abuse has a significant negative association with the mental health of older Indians. The results support the first hypothesis outlined in this paper. Elder abuse may be thought of as a particularly stressful event in later life. Typically Indian parents have continued to invest in their children into adulthood and traditionally have expected to be cared for at an older age. If their investment is not reciprocated, their life is likely to be coloured by a sense of injustice and exploitation, [16] which may lead to certain negative effects such as anger, depressed mood and loneliness. [39] Constant negative effects are known to be compromising to both physical and mental health, with the mechanism of

For peer review only - http://bmjopen.bmj.com/site/about/guidelines.xhtml

pathogenesis operating through physiological changes, including one's immune suppression, and cardiovascular and endocrine reactivity. [19,40] The results suggest that women are more vulnerable than men when encountering abusive behaviours from family members. This might be because those women have fewer psychosocial resources [12] to cope with the negative environment or needed medical assistance as a result of the abuse. Our results are consistent with other empirical studies, suggesting that there is a harmful link between older abuse and psychological health. [27,41,20,21] However, no evidence from this study is found for buffering effects of psychosocial resources, such as social support and perceived ability to control outcomes. The results from this study only highlight a direct and beneficial association between psychosocial resources and psychological health, irrespective of the presence of elder abuse. One possible explanation is that elder abuse is in direct conflict with Indian cultural values, and thus older adults who have been abused may not disclose this information or seek support due to a sense of shame and/or a fear of stigmatization. [42-43] Another explanation may lie in the scale of the outcome variable. In this study, we focus on psychological distress using a nominal scale measurement. Previous studies that have demonstrated the buffering effects of psychosocial resources have measured psychological distress as a ratio scale [27,21] and thus it is possible that by using a nominal scale we may be missing some of the nuances around buffering effects. Interestingly the results in this paper show that household wealth has a direct and inverse relationship with psychological distress, and also offers a substantial link with the relationship between elder abuse and psychological distress. Both qualitative and quantitative studies have found that individuals who have financial or physical assets may feel more in control of their lives, leading to less

For peer review only - http://bmjopen.bmj.com/site/about/guidelines.xhtml

vulnerability to anxiety or mood disorders, or less severe psychological symptoms. [12,44] Unexpectedly, however, we found that the negative association between elder abuse and mental health is significantly stronger among older people living in wealthier households. One possible explanation might be that issues of control over property, finance, and other decisions may result in more family conflict between parents and their adult children or other relatives among wealthier households than in poorer households. This is consistent with qualitative studies in India which have highlighted bitter battles in village families between elders and adult children over land and money. [12,6] Adult children have been reported at times to resent the expense of medical care and treatments for their aged parents, especially when some of the children felt they were providing more than their fair share of the total cost. Again this may be more commonplace amongst wealthier households, where private medical care is an option. Our findings reinforce previous research demonstrating the role of socio-economic circumstances in determining older people's mental health. Poor social and economic circumstances affect individuals' health throughout life. [45-47] The results from this study also add to the evidence base with regard to inequalities in older people's mental health related to levels of physical health [48-51] and different geographic areas, reflecting differences in their social, political, economic arrangements and levels of public health services and social protection.[52]

LIMITATIONS

The present study is limited by the cross-sectional design of the data. It is possible that older people already had stress before the experience of abuse, and at the time of the interview were more likely than others to recall past experiences such as abuse. The results could

For peer review only - http://bmjopen.bmj.com/site/about/guidelines.xhtml

benefit from repeated measures of psychological distress before and after abusive exposures. Another limitation is the self-report nature of the data. There is no validation by agencies charged with investigating elder abuse. Due to social taboo, elder abuse might be underreported; meantime, the interviewer might make an educated guess concerning the presence or absence of physical, emotional and financial abuse or neglect by family members, which may bias our results on the relationship between abuse and distress. Future surveys need to develop appropriate screening and assessment tools to identify elder abuse. [11] Our data also lack purposively designed scales measuring social support and personal coping resources. Future research addressing these issues will improve our understanding of the relationship between elder abuse, psychosocial resources, and psychological distress.

CONCLUSION

In conclusion, the findings presented in this paper demonstrate that 1) in the seven Indian states represented in this research, elder abuse shows a negative association with the mental health of older adults, especially among women; 2) household wealth generally has an inverse relationship with mental health; 3) however, the negative association between elder abuse and mental health is stronger among older people living in wealthy households.

The number of older people in India is steadily growing. [4] Increased life expectancy brings with it more chronic health problems and functional limitations that require long-term care. Most older people continue to live in villages and to experience poor socioeconomic status and are dependent upon their families for both financial and physical support. While the need for care has grown, available resources have decreased. The lack of jobs close to where they

For peer review only - http://bmjopen.bmj.com/site/about/guidelines.xhtml

live forces many young Indians to seek employment in urban areas. Such migration reduces the number of available caregivers and increases the demands on non-migrant family members to shoulder responsibility for elders' care. The costs of care were often high, due to a lack of adequate public health care for older persons. Mistreatment of one's older parents may also emanate from conflict over the control of family property. Researchers have begun to argue that traditional Indian cultural values and the consequences of urbanization and modernization influence the nature and scope of elder abuse. [38] In addition, recent research has highlighted the increasing incidence of elder abuse due to property separation/division, living conditions and the growing generation differences in thinking and attitude towards expectations and lifestyles. [53] As India continues on its path of economic development, with increasing urbanisation and spatial mobility, older people may be further exposed to abuse.

At present, mental health in later life is not a priority area in many low income countries [54] and how it is associated with elder abuse is neglected in both the research and policy arenas. Intergenerational relations between older people and their adult children are pivotal in the health and wellbeing of older people. However, such relations can have both positive and negative impacts upon the mental wellbeing of both the older person and, in certain cases, that of the adult child carer. [55-56] Elder abuse needs to be recognised as a key public health issue, and appropriate strategies, policies and practices put in place. Reducing elder abuse will have a positive impact on both the physical and mental health outcomes in later life. Policy makers in India are faced with a major challenge in a low resource context, however widening the public policy debate to include the recognition of the prevalence of elder abuse and how best to address it within health policy planning would be a key move forward.

For peer review only - http://bmjopen.bmj.com/site/about/guidelines.xhtml

Contributors All authors Maria Evandrou, Jane Falkingham, Min Qin and Athina Vlachantoni contributed to conceptualising, writing, and editing this paper, and have read and approved the final manuscript. Data analysis was performed by Min Qin.

Funding The study was supported under the NWO-ESRC-ICSSR AgeGlobe Network (Indian-European research networking: Ageing and Well-being in a Globalising World; grant number ES/K005979/1).

Competing interests None declared.

Data sharing statement No additional data are available.

For peer review only

References

1 United Nations, Department of Economic and Social Affairs, Population Division, World Population Prospects: The 2015 Revision, Key Findings and Advance Tables. Working Paper No. ESA/P/WP.241, 2015.

2 Dhillon P, Ladusingh L, Agrawal G, Ageing and changing patterns in familial structure for older persons in India: a decomposition analysis. *Quality in Ageing and Older Adults* 2016; 17: 83 – 96. doi: 10.1108/QAOA-10-2014-0024

3 Venkoba RA. Psychiatry of old age in India. *International Review of Psychiatry* 1993; 5: 165–170.

4 Government of India. *Situation Analysis of the Elderly in India*. Central Statistics Office, Ministry of Statistics & Programme Implementation, 2011. http://mospi.nic.in/mospi_new/upload/elderly_in_india.pdf (accessed 14 Oct 2016)

5 Patel V, Prince M. Ageing and mental health in developing countries: who cares? Qualitative studies from Goa, India. *Psychological Medicine* 2001; 31: 29–38. PMID: 11200957

6 Srivastava S, Bhatia MS, Rajoura OP, *et al.* Elder neglect in changing Indian scenario. *Delhi Psychiatry Journal* 2013; 16: 273–276. http://medind.nic.in/daa/t13/i2/daat13i2p273.pdf(accessed 17 Oct 2016)

7 World Health Organization (WHO). *A Global Response to Elder Abuse and Neglect: Building Primary Health Care Capacity to Deal with the Problem Worldwide: Main Report* (pp.1). Geneva: WHO, 2008.

8 Yon YJ, Mikton CR, Gassoumis ZD. et al., Elder abuse prevalence in community settings:

For peer review only - http://bmjopen.bmj.com/site/about/guidelines.xhtml

a systematic review and meta-analysis, *Lancet Glob Health* 2017;5: e147–56.

9 Dong XQ., Elder abuse: systematic review and implications for practice, *J Am Geriatr Soc.* 2015;63(6):1214-38.

10 Pillemer K, Burnes D, Fiffin C, et al., Elder abuse: global situation, risk factors, and prevention strategies, *Gerontologist* 2016; 56 (S2): S194–S205.

11 Momtaz YA, Hamid TA, Ibrahim R. Theories and measures of elder abuse. Psychogeriatrics 2013; 13: 182–188.

12 Soneja S. *Elder Abuse in India* Country Report for World Health Organization.1999. http://www.who.int/ageing/projects/elder_abuse/alc_ea_ind.pdf (accessed 17 Oct 2016)

13 Skirbekk V, James KS. Abuse against elderly in India – the role of education. *BMC Public Health* 2014; 14: 336. http://www.biomedcentral.com/1471-2458/14/336. (accessed 14 Oct 2016)

14 Ohayashi H, Yamada S. *Psychological Distress Symptoms, Causes and Coping*. New York: Nova Science Publishers, Inc. 2012

15 Tyrer P, Steinberg D. *Models for Mental Disorder*. Chichester: John Wiley and Sons. 2013.

16 Winterstein T, Eisikovits Z. The experience of loneliness of battered old women. *Journal of Women & Aging* 2005; 17: 3–19. doi:10.1300/J074v17n04_02

17 Dyer CB, Pavlik VN, Murphy KP, *et al.* The high prevalence of depression and dementia in elder abuse or neglect. *JAGS* 2000; 48: 205-208. PMID: 10682951

18 Begle AM, Strachan M, Cisler JM, *et al.* Elder mistreatment and emotional symptoms

For peer review only

For peer review only - http://bmjopen.bmj.com/site/about/guidelines.xhtml

among older adults in a largely rural population: the South Carolina elder mistreatment study. *Journal of Interpersonal Violence* 2011; 26: 2321–2332. doi: 10.1177/0886260510383037

19 Selye H. Stress and disease. *Science*1955; 122: 625–631. doi: 10.1126/science.122.3171.625

20 Dong XQ, Simon MA, Beck TT, *et al.* Elder abuse and mortality: the role of psychological and social wellbeing. *Gerontology* 2011; 57: 549–558. doi: 10.1159/000321881

21 Luo Y, Waite LJ, Mistreatment and psychological well-being among older adults: exploring the role of psychosocial resources and deficits. *J Gerontol B Psychol Sci Soc Sci,* 2011; 66: 217–229. doi: 10.1093/geronb/gbq096

22 Fisher BS, Regan SL, The extent and frequency of abuse in the lives of older women and their relationship with health outcomes. *Gerontologist* 2006; 46: 200–209. doi: 10.1093/geront/46.2.200

23 Cohen S, Wills TA. Stress, social support, and the buffering hypothesis. *Psychological Bulletin* 1985; 98: 310–357.

24 Lazarus R S, Folkman S, *Stress, Appraisal and Coping*. New York: Springer, 1984.

25 Pearlin LI, Schieman S, Fazio EM, *et al.* Stress, health, and the life course: Some conceptual perspectives. *Journal of Health and Social Behavior* 2005; 46: 205–219. doi:10.1177/002214650504600206

26 Taylor SE, Stanton AL. Coping resources, coping processes, and mental health. *The Annual Review of Clinical Psychology* 2007; 3: 377–401.

For peer review only - http://bmjopen.bmj.com/site/about/guidelines.xhtml

doi:10.1146/annurev.clinpsy.3.022806.091520

27 Comijs HC, Penninx BW, Knipscheer, KP, *et al.* Psychological distress in victims of elder mistreatment: the effects of social support and coping. *J Gerontol B Psychol Sci Soc Sci,* 1999; 54: 240–245. PMID: 12382593

28 Alam M, James KS, Sathyanarayana KM, *et al*. *Report on the status of elderly in select states of India, 2011*. New Delhi: United Nations Population Fund, 2012.

29 Goldberg DP, Gater R, Sartorius N, *et al.* The validity of two versions of the GHQ in the WHO study of mental illness in general health care. *Psychological Medicine* 1997; 27: 191–197. doi: 10.1017/S0033291796004242

30 Shamasunder C, Srram TG, Murali RSG, *et al.* Validity of a short 5 - item version of the General Health Questionnaire (GHQ). *Indian J Psychiatry* 1986; 28: 217–219. PMCID: PMC3172533

31 Shivakumar P, Sadanand S, Bharath S, *et al.* Identifying psychological distress in elderly seeking health care. *Indian J Public Health* 2015; 59: 18–23. doi:10.4103/0019-557X.152849

32 Gautam S, Nijhawan M, Kamal P, Standardisation of Hindi version of Goldberg's General Health Questionnaire. *Indian J Psychiatry* 1987; 29: 63-66. PMID: 21927210

33 Kuruvilla A, Pothen M, Philip K, *et al.* The validation of the Tamil version of the 12 item general health questionnaire. *Indian J Psychiatry* 1999; 41: 217–221. PMCID: PMC2962995

34 Goldberg D, Oldehinkel T, Ormel J, Why GHQ threshold varies from one place to another. *Psychological Medicine* 1998; 28: 915–921. PMID: 9723146

For peer review only - http://bmjopen.bmj.com/site/about/guidelines.xhtml

35 Pearlin LI, Schooler C. The structure of coping. *Journal of Health and Social Behavior* 1978; 22, 337–356. PMID: 649936

36 Sherbourne CD, Stewart AL. The MOS social support survey. *Social Science & Medicine* 1991; 32: 713–714. PMID: 2035047

37 Timmerman IGH, Emanuels-Zuurveen ES, Emmelkamp PMG. The Social Support Inventory (SSI): A Brief Scale to Assess Perceived Adequacy of Social Support. *Clin Psychol Psychother* 2000; 7: 401–410. doi:10.1002/1099-0879(200011)7:5<401::AID-CPP253>3.0.CO;2-I

38 Lwaya T, Doi T, Seichi A, et al. Characteristics of disability in activity of daily living in elderly people associated with locomotive disorders. BMC Geriatrics 2017; 17:165.

39 Martin P, Hagberg B, Poon LW. Predictors of loneliness in centenarians: A parallel study. *J Cross Cult Gerontol* 1997; 12: 203–224. PMID: 14617927

40 Tachè J, Selye H. On stress and coping mechanisms. *Issues in Mental Health Nursing* 1985; 7: 1–4, 3–24. doi: 10.3109/01612848509009447

41 Chokkanathan, S. Resources, stressors and psychological distress among older adults in Chennai, India. *Social Science & Medicine* 2009; 68: 243–250. doi: 10.1016/j.socscimed.2008.10.008

42 Segal U. Family violence: a focus on India. *Aggression and Violent Behavior* 1999; 4: 213–231. doi:10.1016/S1359-1789(97)00051-7

43 Nagpaul K. Elder abuse among Asian Indians: traditional versus modern perspectives. *Journal of Elder Abuse & Neglect* 1998; 9: 77–92. doi: 10.1300/J084v09n02_07

44 Carter KN, Blakely T, Collings S, *et al.* What is the association between wealth and

For peer review only

mental health? *J Epidemiol Community Health* 2008; 63: 221–226. doi:10.1136/jech..079483

45 WHO. *The Solid Facts: Social Determinants of Health*. Copenhagen: World Health Organization Regional Office for Europe, 2003.

46 Gallo LC, Matthews KA, Understanding the association between socioeconomic status and physical health: do negative emotions play a role? *Psychological Bulletin* 2003; 129: 10–51. doi: 10.1037/0033-2909.129.1.10

47 Jeon GS, Jang SN, Rhee SJ, *et al.* Gender differences in correlates of mental health among elderly Koreans. *J Gerontol B Psychol Sci Soc Sci,* 2007; 62B, 5, S323–S329. PMID: 17906176

48 Ormel J, Kempen GIJM, Penninx BWJH, *et al.* Chronic medical conditions and mental health in older people: disability and psychosocial resources mediate specific mental health effects. *Psychological Medicine* 1997; 27: 1065 –1077. PMID: 9300512

49 Joshi K, Kumar R, Avasthi A, Morbidity profile and its relationship with disability and psychological distress among elderly people in Northern India. *Int J Epidemiol* 2003; 32: 978–987. PMID: 14681260

50 Shih M, Simon PA. Health-related quality of life among adults with serious psychological distress and chronic medical conditions. *Qual Life Res* 2008; 17, 521–528. doi:10.1007/s11136-008-9330-9

51 Rajkumar AP, Thangadurai P, Senthilkumar P, *et al.* Nature, prevalence and factors associated with depression among the elderly in a rural South Indian community. *International Psychogeriatrics* 2009; 21: 372–378. doi: 10.1017/S1041610209008527

For peer review only - http://bmjopen.bmj.com/site/about/guidelines.xhtml

52 World Health Organization, Calouste Gulbenkian Foundation (WHO, CGF). *Social Determinants of Mental Health*. Geneva: World Health Organization, 2014.

53 Kumar Y, Bhargava A, Elder abuse in Indian families: problems and preventive actions. *IJSRP* 2014; 4: 1–8. http://www.ijsrp.org/research-paper-1014/ijsrp-p3498.pdf (accessed 14 Oct 2016)

54 Patel V. Mental health in low- and middle-income countries. *British Medical Bulletin* 2007; 81 and 82, 81–96. doi: 10.1093/bmb/ldm010

55 Stone J, Evandrou M, Falkingham J. The transition to living alone and psychological distress in later life. *Age and Ageing* 2013; 42: 366–372. doi:10.1093/ageing/aft006.

56 Vlachantoni A, Robards J, Falkingham J *et al.* Trajectories of informal care and health. *SSM -Population Health* 2016; 2: 495–501. doi:10.1016/j.ssmph.2016.05.009

For peer review only

For peer review only - http://bmjopen.bmj.com/site/about/guidelines.xhtml

Table 1. Distribution of GHQ-12 ≥ 4 (unweighted data)

Variables	Distribution (%)	Number of respondents	% of GHQ-12 score ≥ 4	P value (Pearson Chi square test)
**Total**	100.0	9,589	40.6	
Experience abuse last month				
No	95.5	9,157	39.7	.000
Yes	4.5	432	61.6	
**Selected psychosocial and material resources variables**				
Marital status				
Widowed	40.5	5,710	48.0	.000
Currently married/living together	59.5	3,879	35.7	
Social activities				
No listed social activity	19.4	1,860	55.8	.000
Have social activity	80.6	7,729	37.0	
Have someone trust or confide				
No	17.1	1,642	59.7	.000
Yes	82.9	7,947	36.7	
Feel able to manage situations				
Hardly ever	24.0	2,298	76.3	.000
Sometimes	63.2	6,057	32.8	
Most of the time	12.9	1,234	12.9	
Financial dependency				.000
No dependency	25.3	2,427	25.2	
Partial dependency	24.6	2,357	40.9	
Full dependency	50.1	4,805	48.3	
Household wealth index				
Lowest	20.0	1,915	64.1	.000
Second	20.4	1,958	48.4	
Middle	19.6	1,884	40.4	
Fourth	19.8	1,901	28.8	
Highest	20.1	1,931	21.4	
**Other control variables**				
age				
60-69	63.4	6,082	35.6	.000
70-79	26.4	2,533	46.9	
80+	10.2	974	55.9	
Gender				
Men	47.4	4,543	36.2	.000
Women	52.6	5,046	44.7	
Education				
None	46.1	4,422	52.1	.000
1-4 years	12.9	1,241	45.2	
5-7 years	13.5	1,297	36.9	
8+ years	27.4	2,629	21.0	
Caste				
Scheduled Tribe/Scheduled Caste	24.2	2,316	47.4	.000
Other Backward Caste	34.1	3,274	42.9	
Others	39.1	3,753	33.4	
Unknown	2.6	246	58.5	
Working status				
Has never worked	36.2	3,472	41.2	.000
Has ever worked but not now	40.7	3,904	42.6	
Has ever worked and is now working	23.1	2,213	36.2	
Self-reported health				
Excellent /Very good	16.2	1,557	19.1	.000
Good	30.0	2,875	28.1	
Fair	36.5	3,499	48.7	
Poor	17.3	1,658	65.6	
Chronic disease				
No	35.3	3,389	35.3	.000
1 type	32.2	3,087	41.7	
2 more types	32.5	3,113	45.4	
Difficulty with ADLs				
No need for assistance	92.7	8,890	38.1	.000
Light need	3.8	369	67.8	
Heavy need	3.4	330	79.1	
Disability				
No disability	27.2	2,606	25.1	.000
Light	44.7	4,285	37.6	

For peer review only

Variables	Distribution (%)	Number of respondents	% of GHQ-12 score ≥ 4	P value (Pearson Chi square test)
Medium	18.6	1,788	54.5	
Heavy	9.5	910	72.0	
Living arrangement				
Alone	6.3	605	50.9	.000
Spouse only	14.9	1,432	34.9	
At least one child	71.3	6,833	40.5	
Others	7.5	719	44.6	
Residence				
Rural	52.2	5,001	45.7	.000
Urban	47.8	4,588	35.1	
State				
Himachal Pradesh	15.0	1,440	23.5	.000
Punjab	13.1	1,255	20.8	
West Bengal	13.2	1,263	60.5	
Odisha	15.3	1,467	55.5	
Maharashtra	14.6	1,399	44.3	
Kerala	14.0	1,341	28.0	
Tamil Nadu	14.9	1,424	50.8	

Source: Authors' analysis of UNFPA Building Knowledge Base on Ageing in India 2011 survey

For peer review only

Table 2.Odds ratios for GHQ-12 score ⩾ 4(N=9,589)

	Total			Men			Women		
**Variables**	**Model 1**	**Model 2**	**Model 3**	**Model 1**	**Model 2**	**Model 3**	**Model 1**	**Model 2**	**Model 3**
Experienced abuse in last month No (ref)									
Yes	2.44*** (2.00–2.97)	1.60*** (1.22–2.09)	1.12 (0.53–2.36)	2.14*** (1.58–2.90)	1.42 (0.93–2.16)	0.84 (0.22–3.16)	2.60*** (2.00–3.39)	1.76** (1.23–2.50)	1.33 (0.52–3.38)
**Psychosocial and material resources**									
Marital status Widowed (ref)									
Currently married/living together		1.12 (0.98–1.28)	1.12 (0.98–1.28)		1.29* (1.02–1.63)	1.30 (1.03–1.64)		1.06 (0.89–1.27)	1.07 (0.90–1.27)
Social activities participation No (ref)									
Yes		0.85* (0.74–0.98)	0.85* (0.74–0.97)		1.03 (0.83–1.30)	1.04 (0.83–1.30)		0.77** (0.65–0.91)	0.76** (0.63–0.90)
Have someone trust or confide No (ref)									
Yes		0.69*** (0.60–0.80)	0.70*** (0.60–0.81)		0.65*** (0.52–0.82)	0.67*** (0.53–0.84)		0.71*** (0.59–0.86)	0.71*** (0.58–0.86)
Feel able to manage situations Hardly ever (ref)									
Sometimes		0.25*** (0.22–0.28)	0.25*** (0.22–0.28)		0.25*** (0.21–0.31)	0.25*** (0.21–0.31)		0.24*** (0.20–0.28)	0.24*** (0.20–0.28)
Most of the time		0.12*** (0.09–0.15)	0.12*** (0.09–0.15)		0.12*** (0.09–0.16)	0.11*** (0.08–0.16)		0.11*** (0.08–0.15)	0.12*** (0.09–0.16)
Financial dependency No dependency (ref)									
Partial dependency		1.07 (0.91–1.25)	1.08 (0.92–1.26)		1.05 (0.86–1.29)	1.06 (0.86–1.30)		1.03 (0.80–1.33)	1.04 (0.81–1.35)
Full dependency		1.31*** (1.12–1.53)	1.31** (1.12–1.53)		1.57*** (1.26–1.95)	1.58*** (1.26–1.97)		1.11 (0.88–1.40)	1.12 (0.89–1.42)
Household wealth index Lowest (ref)									
Second		0.83* (0.70–0.97)	0.80* (0.68–0.95)		0.77* (0.61–0.99)	0.74* (0.58–0.96)		0.88 (0.70–1.10)	0.86 (0.68–1.08)
Middle		0.78** (0.65–0.94)	0.73*** (0.61–0.88)		0.97 (0.74–1.27)	0.90 (0.68–1.19)		0.68** (0.53–0.87)	0.64*** (0.49–0.82)
Fourth		0.62*** (0.51–0.76)	0.57*** (0.47–0.70)		0.65** (0.48–0.87)	0.60*** (0.45–0.82)		0.63*** (0.48–0.83)	0.57*** (0.43–0.76)
Highest		0.54*** (0.43–0.67)	0.50*** (0.39–0.62)		0.54*** (0.38–0.76)	0.51*** (0.36–0.72)		0.57*** (0.42–0.78)	0.52*** (0.38–0.71)
**Other control variables**									
Age 60-69 (ref)									
70-79		1.20** (1.06–1.36)	1.20** (1.06–1.36)		1.14 (0.95–1.38)	1.14 (0.95–1.38)		1.25* (1.06–1.48)	1.26** (1.06–1.49)
80+		1.24*	1.25*		1.11	1.13		1.31*	1.32*

For peer review only - http://bmjopen.bmj.com/site/about/guidelines.xhtml

Variables	Total Model 1	Total Model 2	Total Model 3	Men Model 1	Men Model 2	Men Model 3	Women Model 1	Women Model 2	Women Model 3
		(1.03–1.50)	(1.04–1.51)		(0.83–1.48)	(0.84–1.51)		(1.02–1.69)	(1.03–1.70)
Gender Men (ref)									
Women		1.12	1.14						
		(0.95–1.32)	(0.97–1.34)						
Education No schooling (ref)									
1-4 years		0.88	0.88		0.84	0.84		0.88	0.88
		(0.75–1.04)	(0.76–1.04)		(0.66–1.08)	(0.66–1.08)		(0.70–1.09)	(0.71–1.10)
5-7 years		0.86	0.86		0.87	0.87		0.84	0.85
		(0.73–1.01)	(0.73–1.01)		(0.69–1.11)	(0.69–1.11)		(0.67–1.06)	(0.68–1.08)
8+ years		0.59***	0.60***		0.52***	0.53***		0.64***	0.66***
		(0.50–0.69)	(0.51–0.71)		(0.42–0.66)	(0.42–0.67)		(0.50–0.83)	(0.51–0.85)
Caste **Scheduled Tribe/Scheduled Caste (ref)**									
Other Backward Caste		0.91	0.91		0.99	0.99		0.86	0.86
		(0.79–1.06)	(0.79–1.06)		(0.80–1.23)	(0.79–1.23)		(0.70–1.05)	(0.70–1.05)
Others		1.05	1.06		1.06	1.06		1.05	1.06
		(0.91–1.21)	(0.92–1.22)		(0.85–1.31)	(0.86–1.32)		(0.87–1.28)	(0.87–1.29)
Unknown		1.09	1.08		1.23	1.22		1.03	1.03
		(0.78–1.53)	(0.77–1.52)		(0.72–2.09)	(0.72–2.08)		(0.65–1.61)	(0.65–1.62)
Working status Not work (ref)									
Ever work but not now		1.21*	1.23*		1.20	1.20		1.20	1.22*
		(1.02–1.42)	(1.04–1.45)		(0.59–2.45)	(0.59–2.46)		(0.99–1.46)	(1.01–1.49)
Ever work and now		1.17	1.19		1.16	1.15		1.11	1.16
		(0.96–1.42)	(0.97–1.45)		(0.56–2.41)	(0.55–2.40)		(0.85–1.45)	(0.89–1.51)
Self-reported health Excellent/very good/good (ref)									
Fair		2.09***	2.09***		2.10***	2.10***		2.08***	2.09***
		(1.85–2.35)	(1.86–2.36)		(1.76–2.51)	(1.76–2.51)		(1.77–2.45)	(1.78–2.46)
Poor		3.83***	3.84***		4.56***	4.55***		3.47***	3.50***
		(3.24–4.52)	(3.25–4.54)		(3.50–5.94)	(3.49–5.93)		(2.78–4.33)	(2.80–4.36)
Chronic disease No (ref)									
One type		1.17*	1.17*		1.29*	1.29*		1.11	1.11
		(1.03–1.34)	(1.03–1.34)		(1.06–1.58)	(1.06–1.57)		(0.93–1.32)	(0.93–1.32)
More than one types		1.31***	1.31***		1.43**	1.44***		1.23*	1.23*
		(1.13–1.51)	(1.13–1.51)		(1.14–1.78)	(1.15–1.80)		(1.01–1.49)	(1.01–1.49)
Difficulty with ADLs No need for assistance (ref)									
Light need		1.21	1.22		0.85	0.85		1.52*	1.53*
		(0.92–1.60)	(0.93–1.61)		(0.55–1.31)	(0.55–1.31)		(1.06–2.19)	(1.06–2.20)
Heavy need		1.83***	1.84***		2.75***	2.79***		1.44	1.46
		(1.31–2.56)	(1.32–2.58)		(1.51–5.00)	(1.54–5.08)		(0.95–2.19)	(0.96–2.21)
Disability No disability (ref)									
Light		1.55***	1.54***		1.27*	1.25*		1.79***	1.79***

For peer review only - http://bmjopen.bmj.com/site/about/guidelines.xhtml

Variables	Total			Men			Women		
	Model 1	Model 2	Model 3	Model 1	Model 2	Model 3	Model 1	Model 2	Model 3
		(1.35–1.78)	(1.34–1.77)		(1.03–1.56)	(1.02–1.54)		(1.49–2.16)	(1.48–2.16)
Medium		2.23*** (1.87–2.65)	2.22*** (1.87–2.64)		1.81*** (1.39–2.36)	1.79*** (1.37–2.34)		2.56*** (2.03–3.23)	2.57*** (2.03–3.24)
Heavy		3.39*** (2.71–4.25)	3.38*** (2.70–4.23)		2.69*** (1.92–3.77)	2.67*** (1.90–3.75)		4.01*** (2.95–5.44)	4.03*** (2.96–5.48)
Living arrangement Alone (ref)									
Spouse only		0.78 (0.60–1.03)	0.78 (0.59–1.03)		0.65 (0.37–1.13)	0.66 (0.38–1.16)		0.82 (0.57–1.17)	0.81 (0.57–1.17)
At least one child		1.10 (0.87–1.40)	1.10 (0.86–1.39)		0.91 (0.53–1.56)	0.93 (0.54–1.59)		1.14 (0.86–1.50)	1.14 (0.86–1.50)
Others		1.20 (0.89–1.61)	1.19 (0.89–1.60)		1.20 (0.66–2.20)	1.22 (0.67–2.24)		1.13 (0.79–1.60)	1.13 (0.79–1.60)
Residence Rural (ref)									
Urban		0.90 (0.80–1.01)	0.90 (0.80–1.01)		0.77** (0.64–0.91)	0.76** (0.64–0.91)		0.99 (0.85–1.15)	0.99 (0.84–1.15)
State Himachal Pradesh (ref)									
Punjab		0.60*** (0.48–0.74)	0.60*** (0.48–0.75)		0.61** (0.43–0.86)	0.62** (0.44–0.87)		0.56*** (0.42–0.75)	0.56*** (0.42–0.75)
West Bengal		2.63*** (2.13–3.25)	2.60*** (2.10–3.21)		4.23*** (3.06–5.85)	4.25*** (3.07–5.88)		1.78*** (1.34–2.38)	1.74*** (1.30–2.33)
Odisha		2.82*** (2.28–3.49)	2.78*** (2.24–3.44)		3.27*** (2.37–4.50)	3.28*** (2.37–4.53)		2.65*** (1.98–3.56)	2.58*** (1.92–3.47)
Maharashtra		1.69*** (1.36–2.10)	1.67*** (1.35–2.08)		2.16*** (1.56–2.98)	2.17*** (1.57–2.99)		1.37* (1.01–1.85)	1.34 (0.99–1.81)
Kerala		0.96 (0.76–1.20)	0.95 (0.76–1.20)		0.72 (0.50–1.04)	0.73 (0.50–1.05)		1.05 (0.78–1.41)	1.03 (0.77–1.40)
Tamil Nadu		3.81*** (3.01–4.81)	3.75*** (2.97–4.74)		4.63*** (3.24–6.59)	4.62*** (3.24–6.60)		3.17*** (2.31–4.36)	3.07*** (2.23–4.23)
Abuse* Second quintile			1.38 (0.75–2.55)			1.79 (0.68–4.72)			1.32 (0.59–2.95)
Abuse * Middle quintile			2.90** (1.30–6.44)			3.83* (1.07–13.69)			2.72 (0.93–7.91)
Abuse * Fourth quintile			4.31*** (1.87–9.92)			3.36 (0.93–12.18)			5.93** (1.87–18.81)
Abuse * Highest quintile			4.47** (1.67–11.98)			3.45 (0.76–15.74)			5.61* (1.40–22.50)
Abuse * High education			0.74 (0.32–1.70)			0.65 (0.22–1.89)			0.78 (0.17–3.51)
Abuse * Have someone to trust			0.87 (0.44–1.71)			0.72 (0.22–1.89)			0.90 (0.38–2.15)

For peer review only - http://bmjopen.bmj.com/site/about/guidelines.xhtml

Variables	Total Model 1	Total Model 2	Total Model 3	Men Model 1	Men Model 2	Men Model 3	Women Model 1	Women Model 2	Women Model 3
Abuse * Feel able to manage situations some time			0.92 (0.54–1.58)			1.26 (0.53–2.98)			0.80 (0.39–1.60)
Abuse * Feel able to manage situations most time			1.04 (0.42–2.54)			2.43 (0.75–7.90)			0.41 (0.10–1.78)
**Constant**	0.66***	0.60*	0.61*	0.55***	0.51	0.52	0.77***	0.84	0.88
**Cox & Snell R^2**	0.008	0.327	0.328	0.005	0.341	0.342	0.011	0.317	0.319
**Nagelkerke R^2**	0.011	0.441	0.442	0.007	0.467	0.469	0.014	0.424	0.427

Source: Authors' analysis of UNFPA Building Knowledge Base on Ageing in India 2011 survey

***p<0.001, **p<0.01, *p<0.05

Values in the brackets are 95% Confidence intervals

For peer review only

For peer review only - http://bmjopen.bmj.com/site/about/guidelines.xhtml

Figure 1. Predicted probability of GHQ-12>=4 among older adults by elder abuse and household wealth quintile index.

Source: Authors' analysis of UNFPA Building Knowledge Base on Ageing in India 2011 survey

For peer review only

For peer review only - http://bmjopen.bmj.com/site/about/guidelines.xhtml

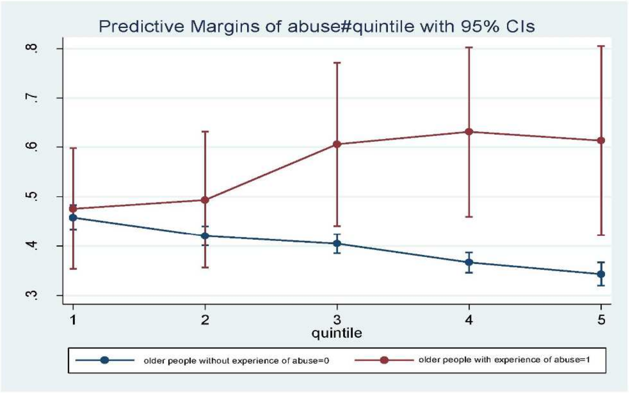

Figure 1. Predicted probability of GHQ-12>=4 among older adults by elder abuse and household wealth quintile
Source: Authors’ analysis of UNFPA Building Knowledge Base on Ageing in India 2011 survey

210x297mm (300 x 300 DPI)

STROBE 2007 (v4) Statement—Checklist of items that should be included in reports of *cross-sectional studies*

Section/Topic	Item #	Recommendation	Reported on page #
**Title and abstract**	1	(*a*) Indicate the study's design with a commonly used term in the title or the abstract	1
		(*b*) Provide in the abstract an informative and balanced summary of what was done and what was found	2,3
**Introduction**			
Background/rationale	2	Explain the scientific background and rationale for the investigation being reported	4,5,6
Objectives	3	State specific objectives, including any prespecified hypotheses	7,8
**Methods**			
Study design	4	Present key elements of study design early in the paper	8
Setting	5	Describe the setting, locations, and relevant dates, including periods of recruitment, exposure, follow-up, and data collection	8
Participants	6	(*a*) Give the eligibility criteria, and the sources and methods of selection of participants	8,9
Variables	7	Clearly define all outcomes, exposures, predictors, potential confounders, and effect modifiers. Give diagnostic criteria, if applicable	9,10,11,12,13
Data sources/ measurement	8*	For each variable of interest, give sources of data and details of methods of assessment (measurement). Describe comparability of assessment methods if there is more than one group	9,10,11,12,13
Bias	9	Describe any efforts to address potential sources of bias	20,21
Study size	10	Explain how the study size was arrived at	9
Quantitative variables	11	Explain how quantitative variables were handled in the analyses. If applicable, describe which groupings were chosen and why	9,10,11,12,13
Statistical methods	12	(*a*) Describe all statistical methods, including those used to control for confounding	14
		(*b*) Describe any methods used to examine subgroups and interactions	14
		(*c*) Explain how missing data were addressed	9
		(*d*) If applicable, describe analytical methods taking account of sampling strategy	-
		(*e*) Describe any sensitivity analyses	-
**Results**			

For peer review only

Participants	13*	(a) Report numbers of individuals at each stage of study—eg numbers potentially eligible, examined for eligibility, confirmed eligible, included in the study, completing follow-up, and analysed	15
		(b) Give reasons for non-participation at each stage	-
		(c) Consider use of a flow diagram	-
Descriptive data	14*	(a) Give characteristics of study participants (eg demographic, clinical, social) and information on exposures and potential confounders	15,16
		(b) Indicate number of participants with missing data for each variable of interest	9
Outcome data	15*	Report numbers of outcome events or summary measures	15
Main results	16	(*a*) Give unadjusted estimates and, if applicable, confounder-adjusted estimates and their precision (eg, 95% confidence interval). Make clear which confounders were adjusted for and why they were included	16,17,18
		(*b*) Report category boundaries when continuous variables were categorized	31,33,34
		(*c*) If relevant, consider translating estimates of relative risk into absolute risk for a meaningful time period	-
Other analyses	17	Report other analyses done—eg analyses of subgroups and interactions, and sensitivity analyses	17,18
**Discussion**			
Key results	18	Summarise key results with reference to study objectives	18,19,20
Limitations	19	Discuss limitations of the study, taking into account sources of potential bias or imprecision. Discuss both direction and magnitude of any potential bias	20,21
Interpretation	20	Give a cautious overall interpretation of results considering objectives, limitations, multiplicity of analyses, results from similar studies, and other relevant evidence	18,19,20
Generalisability	21	Discuss the generalisability (external validity) of the study results	21,22
**Other information**			
Funding	22	Give the source of funding and the role of the funders for the present study and, if applicable, for the original study on which the present article is based	23

*Give information separately for cases and controls in case-control studies and, if applicable, for exposed and unexposed groups in cohort and cross-sectional studies.

Note: An Explanation and Elaboration article discusses each checklist item and gives methodological background and published examples of transparent reporting. The STROBE checklist is best used in conjunction with this article (freely available on the Web sites of PLoS Medicine at http://www.plosmedicine.org/, Annals of Internal Medicine at http://www.annals.org/, and Epidemiology at http://www.epidem.com/). Information on the STROBE Initiative is available at www.strobe-statement.org.

For peer review only
